# A transcriptomics-guided drug target discovery strategy identifies receptor ligands for lung regeneration

**DOI:** 10.1126/sciadv.abj9949

**Published:** 2022-03-23

**Authors:** Xinhui Wu, I. Sophie T. Bos, Thomas M. Conlon, Meshal Ansari, Vicky Verschut, Luke van der Koog, Lars A. Verkleij, Angela D’Ambrosi, Aleksey Matveyenko, Herbert B. Schiller, Melanie Königshoff, Martina Schmidt, Loes E. M. Kistemaker, Ali Önder Yildirim, Reinoud Gosens

**Affiliations:** 1 Department of Molecular Pharmacology, Faculty of Science and Engineering, University of Groningen, Antonius Deusinglaan 1, 9713 AV, Groningen, Netherlands.; 2 Groningen Research Institute for Asthma and COPD, University Medical Center Groningen, University of Groningen, Groningen, Netherlands.; 3 Institute of Lung Biology and Disease (ILBD)/Comprehensive Pneumology Center (CPC), Helmholtz Zentrum München, Member of the German Center for Lung Research (DZL), Munich, Germany.; 4 Aquilo BV, Groningen, Netherlands.; 5 Department of Physiology and Biomedical Engineering, Mayo Clinic College of Medicine, Rochester, MN, USA.; 6 Department of Medicine, University of Pittsburgh, Pittsburgh, PA, USA.

## Abstract

Currently, there is no pharmacological treatment targeting defective tissue repair in chronic disease. Here, we used a transcriptomics-guided drug target discovery strategy using gene signatures of smoking-associated chronic obstructive pulmonary disease (COPD) and from mice chronically exposed to cigarette smoke, identifying druggable targets expressed in alveolar epithelial progenitors, of which we screened the function in lung organoids. We found several drug targets with regenerative potential, of which EP and IP prostanoid receptor ligands had the most profound therapeutic potential in restoring cigarette smoke–induced defects in alveolar epithelial progenitors in vitro and in vivo. Mechanistically, we found, using single-cell RNA sequencing analysis, that circadian clock and cell cycle/apoptosis signaling pathways were differentially expressed in alveolar epithelial progenitor cells in patients with COPD and in a relevant model of COPD, which was prevented by prostaglandin E2 or prostacyclin mimetics. We conclude that specific targeting of EP and IP receptors offers therapeutic potential for injury to repair in COPD.

## Teaser

EP and IP receptors offer novel therapeutic potential for lung repair in COPD.

## INTRODUCTION

One of the main challenges in pharmacology today is the generation of drugs with regenerative potential, with the ability to restore tissue repair in chronic disease. Regenerative medicine has thus far mainly focused on transplantation, tissue engineering approaches, stem or progenitor cell therapy, or a combination of these (*1*). A regenerative pharmacological approach will have considerable additional potential because it can be applied on a relatively large scale. Furthermore, it can be used to halt the disease process in an early stage, resulting in real disease-modifying treatment. In addition, pharmacological targeting may aid or support other regenerative strategies.

There is a need for regenerative pharmacology in respiratory, cardiovascular, and neurological diseases as well as many other disease areas. In respiratory medicine, chronic obstructive pulmonary disease (COPD) is one of the most common lung diseases with a need for regenerative therapies. The disease is characterized by airflow limitation that is not fully reversible and which deteriorates progressively. The main difficulty underlying COPD pathogenesis is increased tissue destruction in combination with abnormal tissue repair in susceptible individuals. As current therapies do not modify the course of the disease, developing new therapeutic strategies aiming at regeneration of tissue is necessary.

In affected individuals, there is an increase in alveolar air space associated with destruction of alveolar epithelial cells along with reduced capacity of epithelial progenitors to restore this defect. In the distal lung, alveolar type II cells and alveolar epithelial progenitors harbor stem cell capacity and function to maintain alveolar epithelium (*2*). These cells reside in a local tissue microenvironment called the niche, which is composed of supporting cells such as fibroblasts and alveolar macrophages. The niche controls adequate activation of the progenitor cell (*1*–*3*) by means of secreted factors such as Wingless-related integration sites (WNTs), fibroblast growth factors (FGFs), retinoic acid, and many other factors that control stemness, proliferation, and differentiation (*3*).

As in many chronic diseases associated with aging, this local lung microenvironment is insufficiently supportive for lung repair in COPD (*1*, *4*, *5*). For example, studies have indicated that an imbalance of canonical and noncanonical WNT signaling results in impaired alveolar regeneration in COPD (*4*, *6*). Moreover, lymphotoxin-β (LTβ), released from CD8^+^ T cells in COPD, can negatively interfere with repair. LTβ induces noncanonical nuclear factor κB signaling, thereby repressing functional Wnt/β-catenin signaling in the lung (*5*). A recent study showed longitudinal changes in lung tissue gene expression associated with repair to underlie disease progression (*7*). Accordingly, the challenge of successful regenerative pharmacology in COPD needs to take into consideration the specific hostile microenvironment and the abnormal repair process that stands in the way of adequate regeneration in COPD.

In the present study, we hypothesized that a transcriptomics-guided drug target discovery strategy based on gene signatures differentially expressed in COPD and in response to cigarette smoke (CS) may be used to identify druggable gene targets that are specifically involved in defective lung repair in COPD. Our results demonstrate that such a strategy coupled to functional studies in organoids yields receptor ligands of which EP and IP prostanoid receptors show the most significant potential in counteracting the negative effects of CS on alveolar progenitor cell function.

## RESULTS

### Transcriptomics-guided screening to identify drug targets

**Fig. 1.**
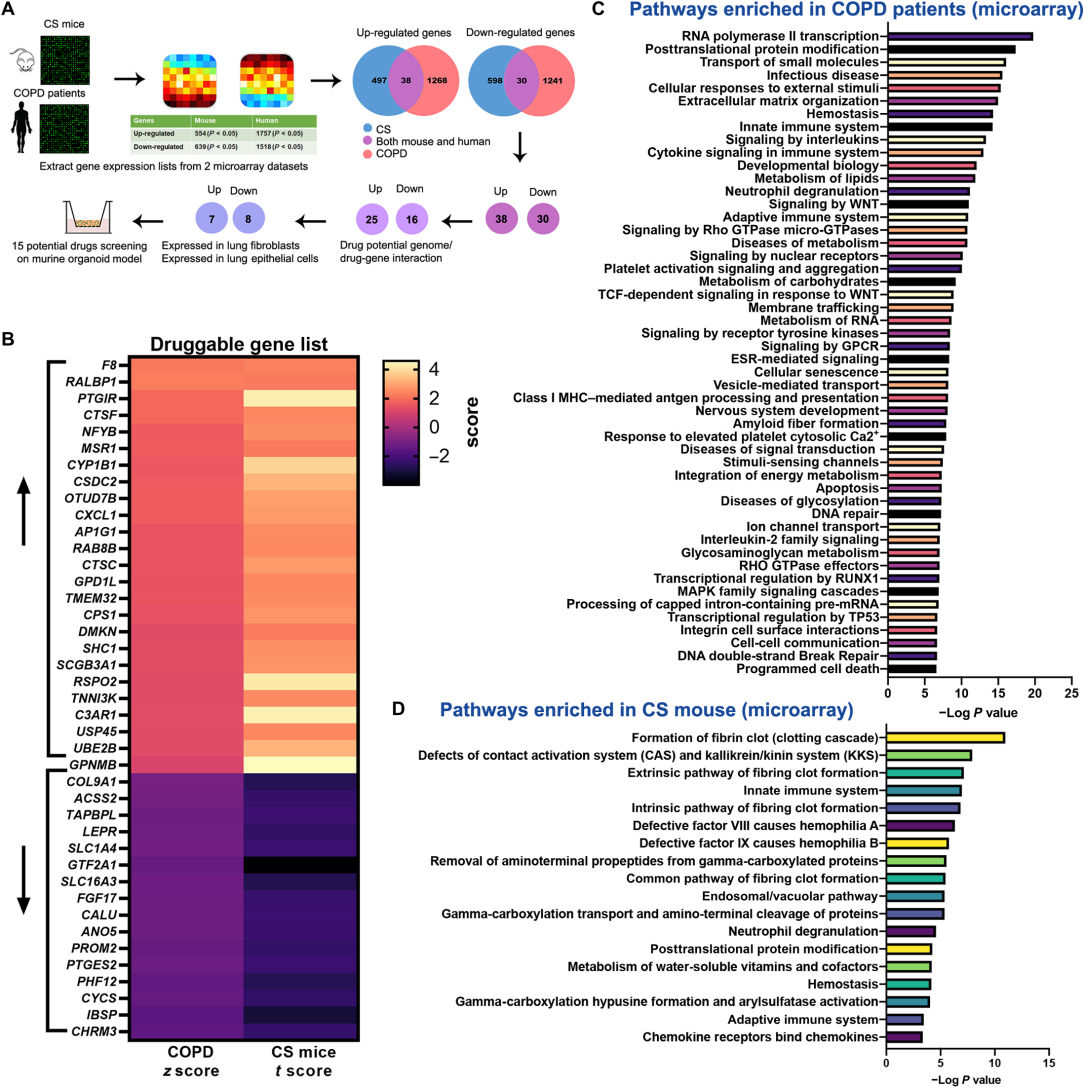
Overview of the transcriptomics-guided drug discovery strategy. (**A**) Schematic outline of the drug screening strategy. (**B**) Heatmap shows the gene expression pattern of the druggable genes (www.dgidb.org) identified both in CS-exposed mice and patient with COPD databases. (**C**) Reactome pathway enrichment analysis of genes differentially expressed from patients with COPD (*8*) using gene set enrichment analysis (GSEA); the top 50 pathways enriched are presented. TCF, T cell factor; GPCR, G protein–coupled receptor; ESR, estrogen receptor; MHC, major histocompatibility complex; RUNX1, runt-related transcription factor 1. (**D**) Reactome pathway enrichment analysis of differentially genes differentially expressed from CS-exposed mice (*9*) using GSEA (www.gsea-msigdb.org/gsea/msigdb/annotate.jsp).

We set out to identify drug targets that may help restore defective lung repair. To achieve this, we used a transcriptomics-guided target discovery strategy (described in Fig. 1A) based on gene signatures of COPD lung tissues (*8*) and of a model of CS exposure (*9*) to identify differentially regulated druggable genes. We found reactome pathways related to inflammation such as neutrophil degranulation and innate immune system to be enriched in both datasets (Fig. 1, C and D) and pathways related senescence, apoptosis, and extracellular matrix regulation to be enriched in COPD, which was also reported in a recent study (*7*) using longitudinal samples. We identified 38 individual target genes that were concordantly up-regulated and 30 individual target genes concordantly down-regulated. These genes were filtered through the “Drug-gene interactions and potential druggability in the Drug Gene Interaction Database” (www.dgidb.org), which rendered 25 druggable up-regulated genes and 16 druggable down-regulated genes (Fig. 1B). Genes were further filtered for expression in lung epithelial cells or fibroblasts by consulting the human Lung Cell Atlas (https://asthma.cellgeni.sanger.ac.uk/) and LungMAP (https://lungmap.net/), which yielded 15 druggable target genes.

**Fig. 2.**
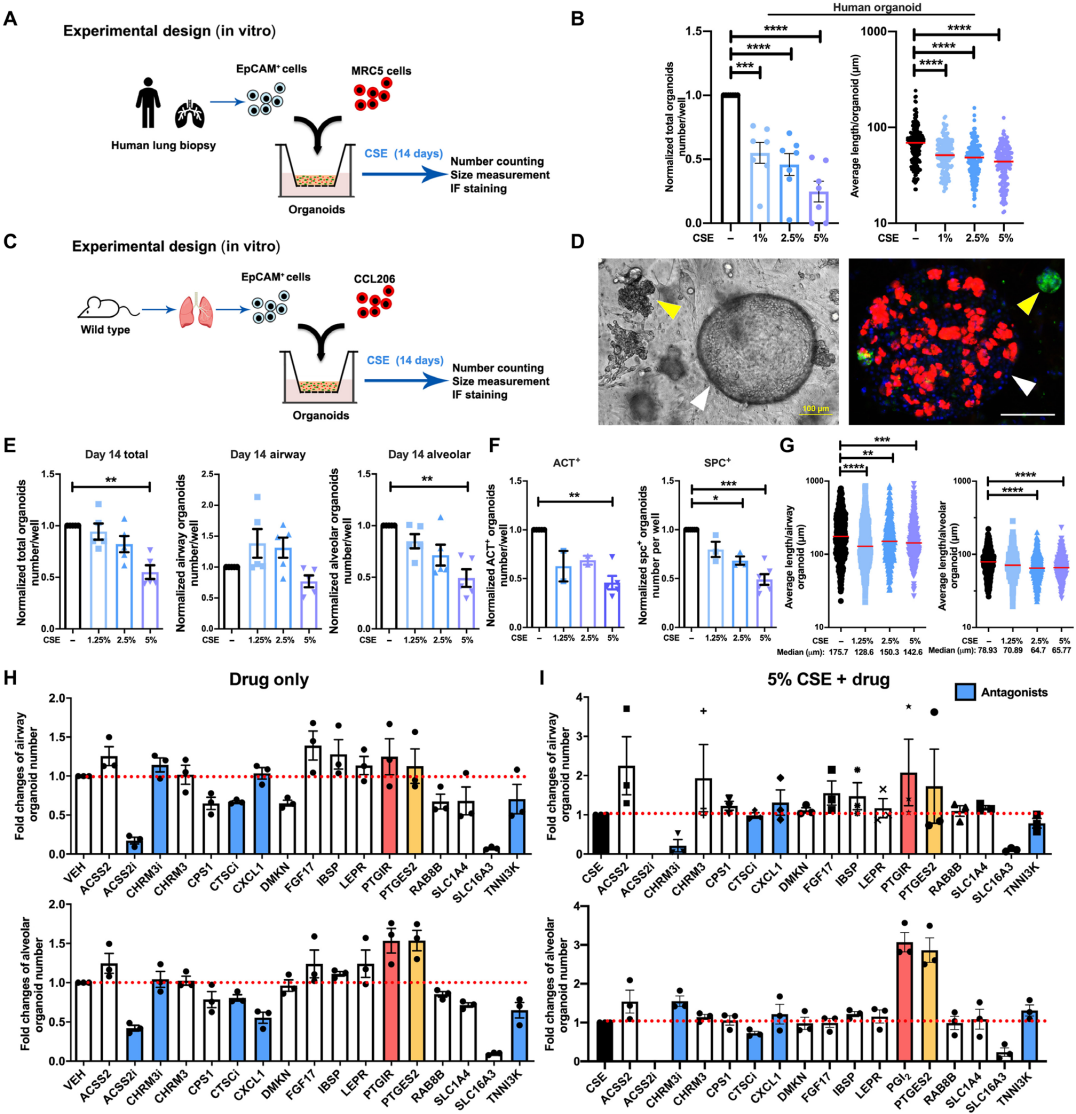
CS exposure represses adult epithelial lung organoid formation. (**A**) Schematic of in vitro human experimental design. (**B**) Quantification of total amount of human organoids and the quantification of average human organoid diameters after treatment with CSE (0, 1, 2.5, and 5%). *N* = 7 experiments (two healthy and five COPD donors), *n* > 150 organoids per group. (**C**) Schematic of in vitro murine experimental design. (**D**) Representative images of murine lung organoids. Left: Light microscopy. Right: Immunofluorescence (IF) of organoids. Green, pro-SPC (SPC); red, ACT; blue, 4′,6-diamidino-2-phenylindole (DAPI). White arrowheads, airway-type organoid; yellow arrowheads, alveolar-type organoid. Scale bars, 100 μm. (**E**) Quantification of the normalized number of total organoids, airway, and alveolar-type organoids on day 14 obtained after treatment with different concentrations of CSE (0, 1.25, 2.5, and 5%). (**F**) Quantification of normalized ACT^+^ and pro-SPC^+^ organoids obtained after treatment with 0, 1.25, 2.5, and 5% CSE. (**G**) Quantification of average organoid diameter after treatment with 0, 1.25, 2.5, and 5% CSE measured on day 14. *N* = 5 experiments, *n* > 380 organoids per group. (**H** and **I**) Overview of drug screening using the in vitro murine lung organoid model. Comparison of the normalized number of airway and alveolar-type organoids treated with the different drugs of interest in the absence (H) or presence (I) of 5% CSE. Red bars, PTGIR; yellow bars, PTGES2; blue bars, antagonists. Data are presented as means ± SEM in number quantification. Data are presented as scatter plots with medians in size quantification. VEH, vehicle; ACSS2, acetate-dependent acetyl CoA synthetase 2; CHRM3, muscarinic M3 receptor; CPS1, carbamoyl phosphate synthetase 1; CTSC, cathepsin C; CXCL1, C-X-C motif chemokine ligand 1; DMKN, dermokine; FGF17, fibroblast growth factor 17; IBSP, integrin binding sialoprotein; LEPR, leptin receptor; PTGIR, prostaglandin I receptor; PTGES2, prostaglandin E synthase 2; RAB8B, Ras-related protein Rab-8B; SLC1A4, solute carrier family 1 member 4; SLC16A3, solute carrier family 16 member 3; TNNIK3, TNNI3 interacting kinase. For all panels, *P < 0.05, **P < 0.01, ***P < 0.001, and ****P < 0.0001.

To assess the potential relevance of signaling functionally associated with the 15 genes of interest, we set up an in vitro organoid model to perform specific drug screening tests. An adult lung epithelial organoid model can recapitulate the in vivo functionality and genetic signature of alveolar epithelium and follow the initial progenitor cell division, subsequent growth, and differentiation of adult lung epithelial cells in a single assay (*6*, *10*). Moreover, the organoid assay allows us to model the interactions between CS extract (CSE) exposure and the alveolar progenitor cell in the most direct possible way. The majority (~70%) of organoids express mature alveolar markers such as pro-SPC (surfactant protein C). Approximately 10% express airway markers [acetylated tubulin (ACT)], and ~10% expressed a mixed phenotype (pro-SPC^+^/ACT^+^) (*10*). Fibroblasts do not integrate into the organoids, which consist entirely of epithelial structures. Thus, we cocultured mouse CD31^−^/CD45^−^/Epcam^+^ (epithelial cellular adhesion molecule^+^) lung epithelial cells with murine CCL-206 lung fibroblasts or human CD31^−^/CD45^−^/Epcam^+^ lung epithelial cells with human MRC5 (Medical Research Council cell strain 5) lung fibroblasts in organoids in Matrigel and exposed these in vitro to different concentrations (1.25, 2.5, and 5%) of CSE (Fig. 2, A and C). The number and size of organoids established by coculturing human lung tissue–derived CD31^−^/CD45^−^/EpCAM^+^ cells and MRC5 fibroblasts were significantly decreased by CSE in a concentration-dependent manner at day 14 (Fig. 2B). The total number of murine organoids quantified at day 14 of treatment with different concentrations of CSE yielded similar results and was decreased in a CSE dose–dependent manner as well (Fig. 2E). To specifically analyze the impact of CSE on organoids derived from alveolar epithelial progenitors, we morphologically subclassified organoids into airway- and alveolar-type (*10*) organoids (Fig. 2D), which revealed that alveolar organoid numbers were more susceptible to CSE exposure than airway organoids (Fig. 2E). Immunofluorescence studies confirmed that the number of ACT (ciliated cell marker, airway-type organoids) and pro-SPC^+^ (type II cell marker, alveolar-type organoids) organoids was significantly decreased by 5% CSE (Fig. 2F). The size of both organoid types was decreased at day 14 (Fig. 2G).

We next aimed to use this lung organoid model to evaluate the efficacy of existing COPD therapeutics. Increasing evidence (*11*, *12*) has linked phosphodiesterase 4 (PDE4) inhibition to the therapeutic management of respiratory diseases, and roflumilast has been used as an oral medication in patients with COPD with a prior history of hospitalization for an acute exacerbation (GOLD 2021). This led us to explore whether the classic PDE4 inhibitor, rolipram, was able to rescue the CS-induced reduction in organoid formation by alveolar progenitors. Thus, organoids were subjected in vitro to different concentrations (1 and 10 μM) of rolipram in the presence and absence of 5% CSE for up to 14 days (fig. S1A). Rolipram (10 μM) alone significantly increased the total number of organoids at day 7 and the pro-SPC^+^ organoids at day 14 (fig. S1, B and C) but had no beneficial effects on organoid numbers when combined with CSE exposures. Treatment with rolipram (1 μM) either alone or in combination with CSE induced significantly increase alveolar organoid size (fig. S1D). The combination of rolipram and olodaterol tended to restore the defective organoid formation under the exposure of 5% CSE (fig. S1, K to M).

Budesonide is an inhaled corticosteroid used in COPD management (*13*–*16*). Therefore, we examined its effect also in our in vitro (fig. S1E) and in vivo (fig. S1H) CS/organoid models. Budesonide (1, 10, and 100 nM) in combination with CSE exposure further reduced the number and the size of both airway- and alveolar-type organoids as compared to CSE exposure alone (fig. S1, F and G). In vivo exposure to budesonide together with CS increased the number of airway but not alveolar organoids (fig. S1I), without affecting the organoid size (fig. S1J). Together, these data show that in vitro exposure to CSE functionally represses human alveolar epithelial progenitor organoid formation, resulting in reduced growth and differentiation, which can be mimicked using murine alveolar epithelial progenitors. Validating the limitations of current pharmacology, PDE4 inhibitors and corticosteroids do not prevent or reduce the detrimental effects of CS on organoid formation.

The assay was used subsequently to screen for the functionality of the targets in restoring organoid growth. Genes down-regulated in response to CS and COPD were targeted with activating ligands or with ligands mimicking an active state of the protein, whereas genes up-regulated in response to CS and COPD were targeted using antagonists, with the exception of *ACSS2* and *CHRM3* for which we included both an agonist and an antagonist. In addition, we included an agonist of *PTGIR* because of its previously suggested beneficial effects in COPD (*17*). The effects of the drugs targeting the 15 selected genes alone (compared to vehicle; Fig. 2H) and in the presence of 5% CSE exposure (Fig. 2I) on the number of organoids were determined. Specific information of all drug effects on organoid number and size are summarized in figs. S2 and S3. The compound activating ACSS2 [acetyl coenzyme A (CoA) synthetase short-chain family member 2] increased the number of airway-type organoids and the size of alveolar organoids in combination with CSE (figs. S2 and S3), whereas the ACSS2 inhibitor had the opposite effects. Atropine [muscarinic M3 receptor (CHRM3) antagonist], IBSP (integrin binding sialoprotein), and LEPR (leptin receptor) tended to increase the number and size of alveolar organoids in response to CSE as well (figs. S2 and S3). However, considering the overall magnitude of alveolar-type organoids, particularly, 16,16-dimethyl prostaglandin E2 (PGE_2_) (stable analog of PGE_2_, the enzymatic product of target gene *PTGES*) and iloprost [prostacyclin (PGI_2_) analog, ligand for target gene *PTGIR*] were identified as the by far most promising targets with regards to their capacity in restoring the CSE-induced repression of organoid formation (Fig. 2, H and I, and fig. S5).

### PGE_2_ and PGI_2_ significantly prevent alveolar epithelial dysfunction

**Fig. 3.**
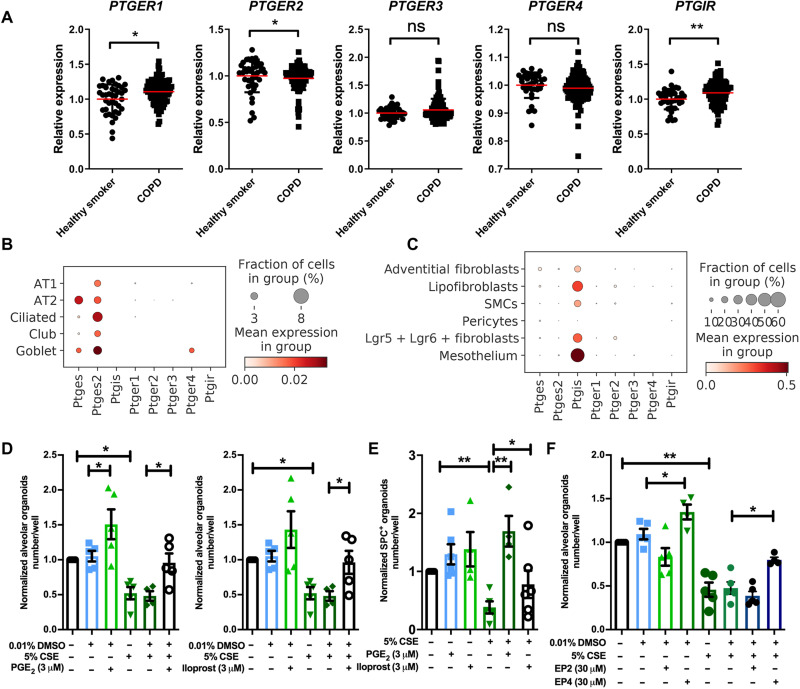
16,16-dimethyl PGE_2_, iloprost, and selective EP2 and EP4 analogs restore lung organoid formation in response to CS(E). (**A**) The relative gene expression of *PTGER1*, *PTGER2*, *PTGER3*, *PTGER4*, and *PTGIR* in healthy smokers (*N* = 40) and patients with COPD (*N* = 111) downloaded from the National Center for Biotechnology Information (NCBI) Gene Expression Omnibus (GEO) database (GSE76925). (**B** and **C**) Data are extracted from the NCBI GEO database (GSE151674). (B) The expression *of Ptges*, *Ptges2*, *Ptgis*, *Ptger1*, *Ptger2*, *Ptger3*, *Ptger4*, and *Ptgir* in epithelial cells using scRNA-seq analysis of mouse lung tissue. (C) The expression *of Ptges*, *Ptges2*, *Ptgis*, *Ptger1*, *Ptger2*, *Ptger3*, *Ptger4*, and *Ptgir* in mesenchymal cells using scRNA-seq analysis of mouse lung tissue. SMCs, smooth muscle cells. (**D**) Quantification of normalized number of alveolar-type organoids treated with vehicle control or 5% CSE ± PGE_2_ agonist (16,16-dimethyl PGE_2_)/iloprost. DMSO, dimethyl sulfoxide. (**E**) Quantification of normalized number of SPC^+^ organoids treated with vehicle control or 5% CSE ± PGE_2_ agonist (16,16-dimethyl PGE_2_) or iloprost. (**F**) Quantification of normalized number of alveolar-type of organoids treated with vehicle control or 5% CSE ± selective EP2 or EP4 agonist. Data are presented as means ± SEM. *P < 0.05, **P < 0.01, ***P < 0.001, and ****P < 0.0001. ns, not significant.

The *PTGES2* and *PTGIR* genes encode membrane-associated PGE synthase and the PGI_2_ receptor, respectively. PGE_2_ acts on four receptor subtypes, being *PTGER1* to *PTGER4*, whereas PGI_2_ acts primarily on *PTGIR*. We assessed their expression in human lung tissue of healthy smokers and patients with COPD (GSE76925) and found maintained expression of all receptors in disease with some small differences in expression, most notably a reduced expression of *PTGER2* and increased expression of *PTGIR* (Fig. 3A). Single-cell RNA sequencing (scRNA-seq) data from human lung tissue shows similar expression of all five receptors in alveolar epithelial cells and in fibroblasts (www.copdcellatlas.com) (*18*). scRNA-seq of mouse lung tissue shows that expression of *Ptger2* and *Ptger4* were the highest compared to that of *Ptger1* and *Ptger3* in mesenchymal cells (Fig. 3, B and C). The expression of *PTGES* and *PTGES2*, the enzymes responsible for PGE_2_ synthesis, was relatively ubiquitous in human and mouse lung tissue, whereas *PTGIS*, the enzyme responsible for PGI_2_ synthesis, was the highest in mesenchymal cell types including fibroblasts.

**Fig. 4.**
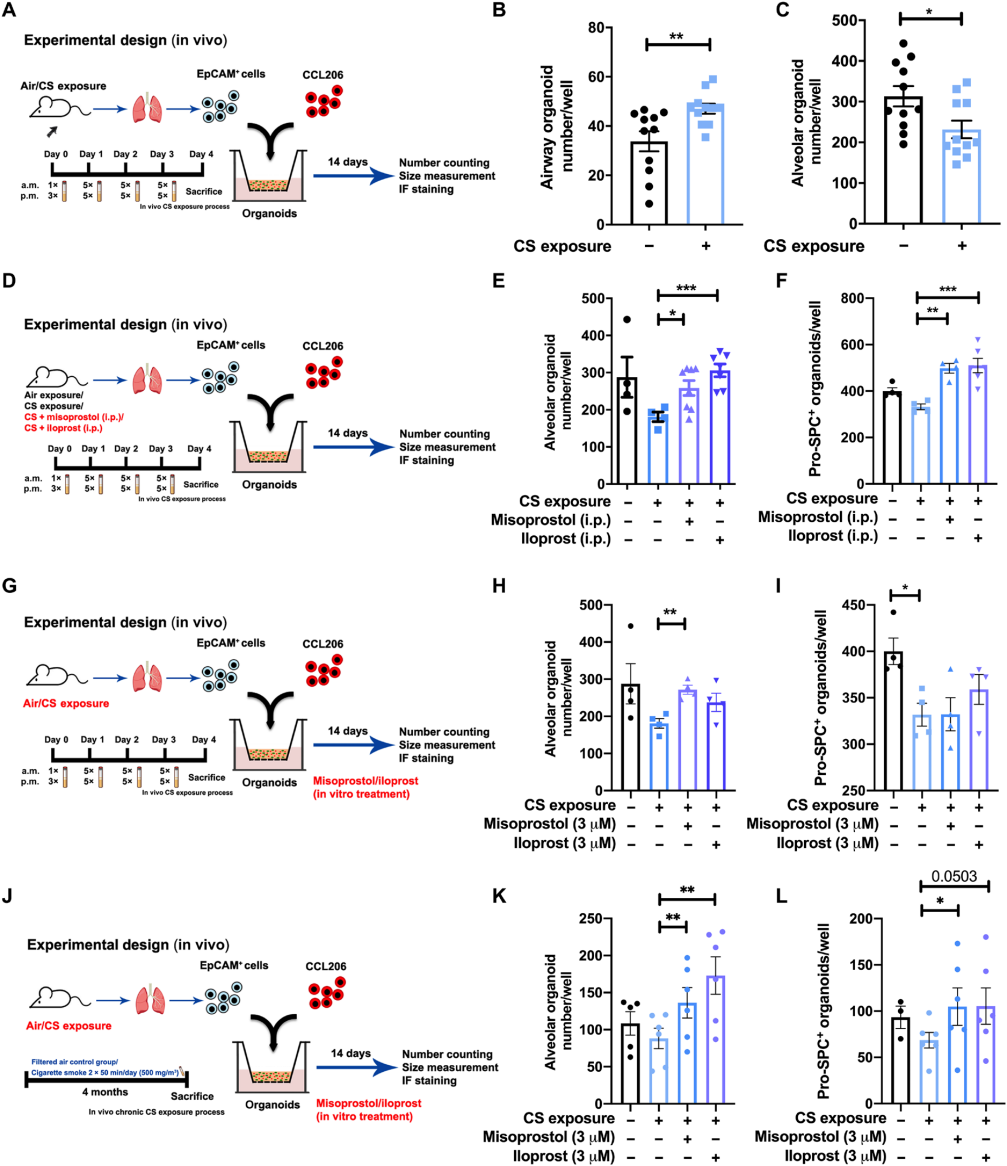
Administration (in vivo and in vitro) of misoprostol and iloprost to CS-exposed mice restored lung organoid formation. (**A**) Schematic of in vivo CS exposure experimental setup. (**B** and **C**) Number of airway- and alveolar-type organoids quantified on day 14 of coculturing CCL-206 fibroblasts and Epcam^+^ cells (isolated from air-exposed/CS-exposed mice). *N* = 11 experiments. (**D**) Schematic of experimental design. Organoids were generated from air- or CS-exposed mice treated in vivo with misoprostol [intraperitoneal (i.p.)] or iloprost (intraperitoneal); all organoids were treated with normal organoid medium. (**E** and **F**) Number of alveolar-type and pro-SPC^+^ organoids quantified on day 14 from coculture of CCL-206 fibroblasts and Epcam^+^ cells [isolated from air-exposed (control) and CS-exposed mice treated intraperitoneally with misoprostol or iloprost]. (**G**) Schematic of experimental design. Organoids were generated from mice exposed to air or CS. Misoprostol and iloprost were added in vitro to the organoid medium for treatment. (**H** and **I**) Number of alveolar-type and SPC^+^ organoids quantified on day 14 from coculture of CCL206 fibroblasts and Epcam^+^ cells (isolated from air- and CS-exposed mice) treated with misoprostol/iloprost in vitro. (**J**) Schematic of experimental design. (**K** and **L**) Number of alveolar-type and SPC^+^ organoids quantified on day 14 from coculture of CCL-206 fibroblasts and Epcam^+^ cells (isolated from air- and CS-exposed mice for 4 months) treated with misoprostol/iloprost in vitro. Data are presented as means ± SEM. *P < 0.05, **P < 0.01, and ***P < 0.001.

To further characterize the effects of PGE_2_ and PGI_2_ on defective alveolar epithelial progenitors, we examined them in vitro (Fig. 3, D to F) and in vivo (Fig. 4) CS(E). The PGE_2_ analog 16,16-dimethyl PGE_2_ and the PGI_2_ analog iloprost both increased the number of alveolar-type organoids even in the presence of 5% CSE (Fig. 3D) and significantly increased the number of SPC^+^ organoids under conditions of CSE exposure (Fig. 3E). To address the relative roles of the two G_s_-coupled PGE_2_ receptors, EP2 and EP4, we evaluated the selective agonists [(R)-butaprost and 5-[(3*S*)-3-hydroxy-4-phenyl-1-buten-1-yl]1-[6-(2*H*-tetrazol-5R-yl)hexyl]-2-pyrrolidinone; CAY10598] of these receptors. Focus was on these G_s_-coupled receptors, as we found that cholera toxin, a well-known inducer of constitutive adenylyl cyclase activity and adenosine 3′,5′-monophosphate (cAMP) signaling, increased organoid formation both with and without the exposure to CSE (fig. S4A). The EP2-selective butaprost had no effect on organoid number (Fig. 3F) but increased the alveolar size in the absence and presence of 5% CSE (fig. S5C). The EP4-selective agonist increased the number of alveolar-type organoids significantly and prevented the number reduction resulting from 5% CSE exposure (Fig. 3F). The EP4 agonist also increased the size of both types of organoids in the absence and presence of 5% CSE (fig. S5C). To explore whether the duration of drug exposure affected organoid formation, we treated the organoids in vitro with PGE_2_ or PGI_2_ analog for three different time windows during organoid development as illustrated in fig. S5 (D to G). These time windows were identified previously (*10*) and mark the initial division phase (days 0 to 2), proliferation (days 2 to 7), and differentiation phase (days 7 to 14). We observed no effect on the number of organoids for any of the short-term drug treatments, suggesting that continuous treatment with or iloprost during all phases of organoid formation is required.

To examine the effects of PGE_2_ and PGI_2_ in vivo, we exposed mice to air (vehicle control), CS, CS + misoprostol (PGE_2_ analog), or CS + iloprost as shown in Fig. 4. For the in vivo studies, we diverted to misoprostol as PGE_2_ analog, as this is a well-tolerated, safe analog of PGE_2_ that is also used clinically. To assess the impact of in vivo CS exposure to the apical epithelial side only and to investigate the immediate impact of CS on the alveolar epithelial progenitors, we exposed mice to air or CS for 1 week, subsequently isolated CD31^−^/CD45^−^/Epcam^+^ cells, and cocultured these with CCL-206 fibroblasts in vitro for 14 days (Fig. 4A). The number of alveolar organoids was significantly decreased after in vivo CS exposure, indicating that a relatively short exposure to CS in vivo is sufficient to capture early changes in progenitor cell function (Fig. 4C). CS exposure did not change the yield of CD31^−^/CD45^−^/Epcam^+^ cells, whereas exposing mice to misoprostol or iloprost increased the yield of Epcam^+^ cells (fig. S6A). The organoid assay revealed that in vivo (intraperitoneal) treatment with either misoprostol or iloprost significantly increased the number of alveolar-type and SPC^+^ organoids (Fig. 4, E and F) ex vivo. Next, to investigate whether in vitro drug treatment would have similar effects on damage caused by in vivo CS exposure, we isolated Epcam^+^ cells from either air- or CS-exposed mice and subjected these to in vitro misoprostol or iloprost treatment in the organoid assay for 14 days (Fig. 4G). In vitro misoprostol increased the number of alveolar-type organoids in cultures derived from CS-exposed mice (Fig. 4H). Only in vitro misoprostol increased the size of alveolar organoids derived from CS-exposed animals (fig. S6C). To validate these findings in a chronic CS model, we isolated CD31^−^/CD45^−^/ Epcam^+^ cells from mice exposed to CS for 4 months and subjected these cells in vitro to misoprostol or iloprost treatment in the organoid assay for 14 days. In vitro misoprostol and iloprost increased the number of alveolar-type organoids in cultures derived from chronically CS-exposed mice to the same extent as what we observed for the 1-week exposure model (Fig. 4K). Misoprostol also increased the number of SPC^+^-differentiated organoids (Fig. 4L). Together, our data show that PGE_2_ and PGI_2_ analogs protect alveolar epithelial progenitor function from the effects of CS exposure. In addition, EP4 rather than EP2 seems to mediate the protective effects of PGE_2_.

### Distinct genetic signatures in regulation of defective alveolar epithelial repair

**Fig. 5.**
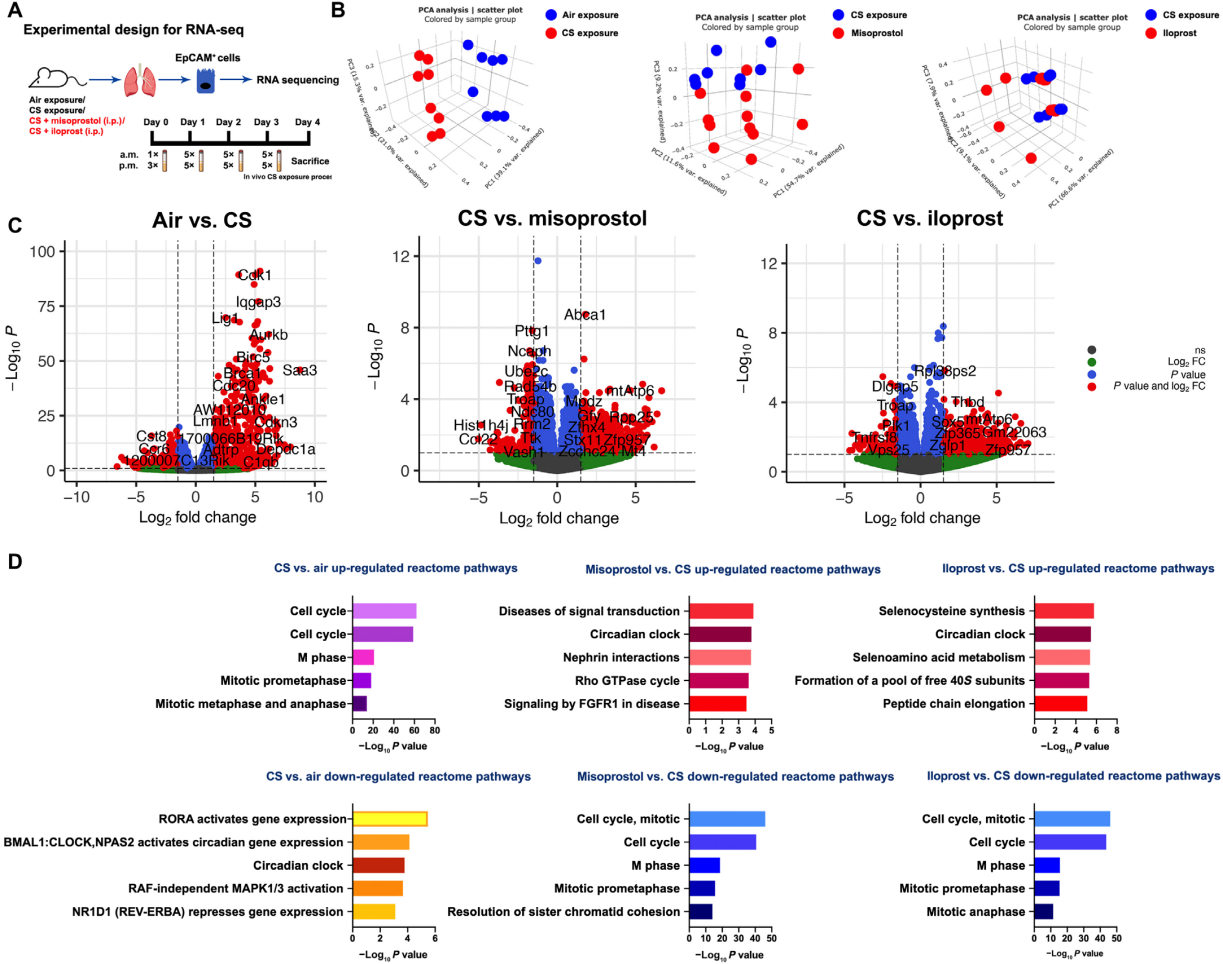
Transcriptomic signatures in response to CS with(out) misoprostol and iloprost. (**A**) Schematic experimental design for RNA-seq. (**B**) PCA plots demonstrate the clusters between different comparisons: air versus CS, CS versus CS + misoprostol, and CS versus CS + iloprost. (**C**) Volcano plots displaying the differentially expressed genes with log_2_ fold change (FC) of at least one with their corresponding *P* values. The entire list of differently expressed genes is provided in the Supplementary Materials. (**D**) The top five significantly up- and down-regulated reactome pathways enrichment form differentially expressed genes within the comparisons of air versus CS exposure, CS exposure versus CS + misoprostol, and CS exposure versus CS + iloprost. The top 20 significantly enriched pathways are shown in table S3. RORA, RAR-related orphan receptor alpha; BMAL, brain and muscle ARNT-like 1; CLOCK, circadian locomotor output cycles kaput; NPAS2, neuronal PAS Domain Protein 2; RAF, rapidly accelerated fibrosarcoma.

To unravel the transcriptional changes leading to impaired lung organoid formation after exposure to CS and the mechanisms underlying the beneficial effects of PGE_2_ and PGI_2_ treatment, we performed RNA-seq (Fig. 5A) on Epcam^+^ cells isolated from mice exposed to air (control), CS, CS + misoprostol (intraperitoneal), or CS + iloprost (intraperitoneal) directly after the isolation procedure (i.e., before inclusion in the organoid assay). Principal component analysis (PCA) revealed that the CS-exposed group is transcriptionally distinct from the control group (Fig. 5B) and that the CS + misoprostol and CS + iloprost groups are transcriptionally different from the CS-exposed group. The top differentially expressed genes from these three comparisons, including both up- and down-regulated genes, are shown in the volcano plot (Fig. 5C) and summarized in the Supplementary Materials. Reactome pathway analysis was used to identify molecular pathways overrepresented in CS/misoprostol/iloprost-modulated genes in alveolar epithelial cells. Within the top 20 enriched pathways (Fig. 5D and the Supplementary Materials), genes associated with cell cycle, mitotic prometaphase, DNA replication/synthesis, and RHO guanosine triphosphatases (GTPases) activating formins signaling pathways were up-regulated by CS exposure compared to air (control) exposure; however, these were down-regulated by treatment with misoprostol or iloprost. Notably, genes associated with the circadian clock signaling pathway were down-regulated by CS exposure but restored by treatment with misoprostol or iloprost. Moreover, signaling by FGF receptor 1 (FGFR1), FGFR3, and FGFR4 were down-regulated in response to CS exposure, whereas the same signaling pathways were up-regulated by misoprostol but not iloprost treatment. These findings suggest that the repair mechanisms of misoprostol and iloprost in response to CS include cell cycle and circadian clock signaling and that the distorted FGFR signaling resulting from CS was corrected only by misoprostol. In addition, the nuclear receptor transcription pathway, growth hormone receptor signaling, mitogen-activated protein kinase (MAPK), WNT, and cell-cell communication signaling pathways appear to be down-regulated in response to CS, but not in either the misoprostol or iloprost treatment group. Overall, the RNA-seq analysis demonstrates both common and distinct transcriptomic mechanisms of misoprostol and iloprost treatment in response to CS exposure in alveolar epithelial progenitors.

### Circadian clock signaling and alveolar epithelial repair

**Fig. 6.**
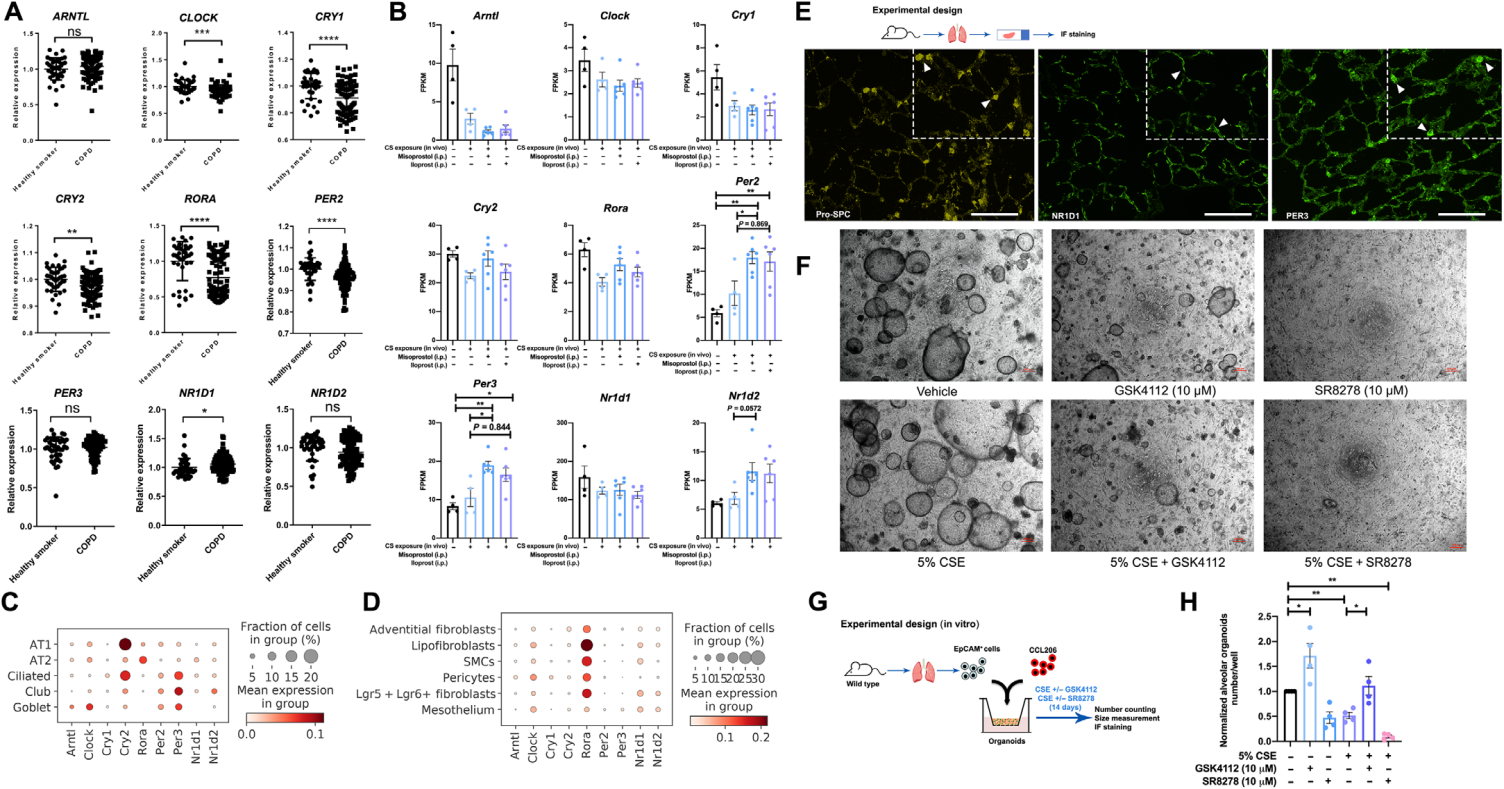
Circadian clock signaling in healthy and diseased lung tissue. (**A**) The relative genes expression of *ARNTL* (*BMAL1*), *CLOCK*, *CRY1*, *CRY*2, *RORA*, *PER2*, *PER3*, *NR1D1*, and *NR1D2* in healthy smokers (*N* = 40) and patients with COPD (*N* = 111) downloaded from the NCBI GEO database (GSE76925). (**B**) The expression of *Arntl* (*Bmal1*), *Clock*, *Cry1*, *Cry2*, *Rora*, *Per2*, *Per3*, *Nr1d1*, and *Nr1d2* genes in air-, CS-, CS + misoprostol–, and CS + iloprost–exposed epithelial cells. FPKM, fragments per kilobase of transcript per million mapped reads. (**C** and **D**) The expression of *Arntl* (*Bmal1*), *Clock*, *Cry1*, *Cry2*, *Rora*, *Per2*, *Per3*, *Nr1d1*, and *Nr1d2* in epithelial cells and mesenchymal cells using scRNA-seq of mouse lung tissue (GSE151674). (**E**) Immunofluorescence staining of pro-SPC (yellow), NR1D1 (green), and PER3 (green) on lung sections acquired from wild-type mice. Scale bars, 50 μm. (**F**) Schematic of experimental design. Scale bars, 200 μm. (**G**) Representative images of lung organoids treated with vehicle control or 5% CSE ± GSK4112 (10 μM)/SR8278 (10 μM). (**H**) Quantification of normalized number of alveolar-type of organoids treated with vehicle control or 5% CSE ± GSK4112 (10 μM)/SR8278 (10 μM). Data are presented as means ± SEM. *P < 0.05, **P < 0.01, ***P < 0.001, and ****P < 0.0001.

Having demonstrated that the circadian clock signaling pathway was down-regulated by CS exposure and restored by in vivo treatment of misoprostol or iloprost, we next set out to explore this finding in more detail. We assessed the mRNA expression of core circadian clock genes in the lung tissue from patients with COPD (GSE76925) and found that the expression of *CLOCK*, *CRY1*, *CRY2*, *RORA*, and *PER2* were all significantly decreased in COPD (Fig. 6A). We next assessed these same transcriptional nine core circadian clock genes in the mouse lung epithelial cells we obtained after in vivo CS exposure and misoprostol/iloprost treatment (described in Fig. 5A). The gene expression of these clock genes was mostly down-regulated in CS-exposed alveolar epithelial progenitors (Fig. 6B). Misoprostol and iloprost increased the expression of *Per2*, *Per3*, and *Nr1d2* in particular (Fig. 6B). scRNA-seq of mouse lung tissue showed that these nine core circadian clock genes were mainly expressed in alveolar epithelial cells and fibroblasts (Fig. 6, C and D, and fig. S7). In particular, the expression of *Arntl*, *Clock*, *Cry2*, *Rora*, *Per3*, and *Nr1d1* were highly expressed in alveolar epithelial cells (Fig. 6C), whereas *Clock*, *Rora*, and *Nr1d1* were highly expressed in the fibroblasts (Fig. 6D). Among epithelial cell types, the core circadian clock gene *Rora* was mainly expressed in alveolar epithelial type II cells; among mesenchymal cell types, it was mainly expressed in leucine-rich repeat-containing G protein-coupled receptor 5^+^/6^+^ (Lgr5^+^/Lgr6^+^) fibroblasts (Fig. 6, C and D, and fig. S7).

The nuclear heme receptor Rev-erbα, encoded by *NR1D1*, plays an essential role in clock-dependent lung physiology and anti-inflammatory response (*19*–*21*). In line with these findings, the immunofluorescence staining of mouse lung tissue showed that NR1D1 (nuclear receptor subfamily 1, group D, member 1) and PER3 (period circadian clock 3) were expressed at the protein level in alveolar epithelial cells and PER3 was particularly enriched in alveolar epithelial type II cells (Fig. 6E). Next, to functionally evaluate the role of Rev-erbα in defective lung repair, we used an agonist and antagonist of Rev-erbα (Fig. 6, F and G). The Rev-erbα agonist GSK4112 (10 μM) increased the number of alveolar-type organoids, both compared to vehicle and in the presence of 5% CSE (Fig. 6H). Conversely, the Rev-erbα antagonist SR8278 (10 μM) significantly prevented organoid formation (Fig. 6H), both in the absence and in the presence of 5% CSE.

## DISCUSSION

COPD results from repeated microinjuries to the epithelium often caused by tobacco smoking. In susceptible individuals, this results in tissue remodeling in the conducting airways but destruction of the respiratory bronchioles and alveoli (*22*). Such a disparity was also found in our organoid assay, which revealed higher numbers of airway organoids but lower numbers of alveolar organoids in response to in vivo CS exposure. No clinically approved pharmacological treatment prevents or reverses the tissue destruction in the distal lung. The results of our study are in line with this contention and demonstrate that the PDE4 inhibitor rolipram and the corticosteroid budesonide had no, or only very limited, beneficial effects on impaired organoid growth and differentiation in response to CSE in vitro or in response to CS exposure in vivo. If anything, budesonide appeared to restrict progenitor cell growth, which is a concern, given the wide use of corticosteroids in the management of COPD. These data underscore the need for novel drug targets.

Consequently, we set out to search for new potential drug targets for lung repair in COPD and identify EP and IP receptor agonists as two such potential targets using a transcriptomics-guided drug discovery strategy. Both EP and IP receptor agonists were able to promote epithelial repair responses after exposure to CS(E). Whereas PGE_2_ and PGI_2_ showed the most profound changes, other methods including ACSS2 agonism, LEPR agonism, and IBSP agonism yielded smaller effects. ACSS2 supports acetyl-CoA synthesis from acetate in the cytosol (*23*, *24*) and thereby plays an important role in lipid metabolism and in the regulation of histone acetylation in the nucleus during gene transcription. IBSP is a member of the small integrin-binding ligand *N*-linked glycoprotein family (*25*, *26*), which is associated with bone metastases of lung cancer (*27*). LEPR is an adipocytokine that not only has a central role in regulating food intake and energy expenditure (*28*) but also has been linked to lung function decline in a population in COPD (*29*). Nonetheless, among all the candidate targets, PGE_2_ and PGI_2_ analogs emerged as the most promising compounds among all drugs in the current study. PGs (prostaglandins) are lipid mediators synthesized from arachidonic acid via the cyclooxygenase pathway and include PGD2, PGI_2_, PGH2, PGE_2_, and PGF2α (*30*). PGI_2_ signals via IP receptors to induce cAMP signaling, similar to EP4 receptors. We show EP4 receptors to have similar expression as in non-COPD controls, whereas IP receptors are expressed at higher levels in patients with COPD, indicating that the expression of both receptors is maintained in disease.

We show that PGE_2_ agonists are beneficial in reducing CS-induced damage to alveolar epithelial progenitors. However, PGE_2_ has been reported as an unstable molecule with an extremely short half-life; therefore, targeting its receptors with specific more stable analogs may be a better alternative. PGE2 is the most widely produced PG in the human body, and it signals via four specific G protein–coupled receptors (EP1 to EP4) (*31*, *32*). The interactions between PGE_2_ and EP receptors depend on tissue and cell type, specific receptor expression, and differences in binding affinities, leading to unique patterns of EP receptor activation (*33*). PGE_2_ can stimulate cAMP production through EP2 and EP4 receptors, whereas EP3 activation results in decreased cAMP synthesis and EP1 stimulation is coupled to G_q_ activation and (enhanced) Ca^2+^ signaling (*30*, *33*, *34*). EP1 and EP3 receptors can mediate bronchoconstriction indirectly through activation of neural pathways (*35*), as a consequence nonselective PGE_2_ analogs are unsuitable as pulmonary drugs. Therefore, we selected analogs of EP2 and EP4 to mimic effect of PGE_2_ in our organoid assay and demonstrated that EP4 agonism showed beneficial effects against impaired organoid formation in response to CS(E) exposure. Targeting EP4 receptors is worthwhile investigating in more detail in the future, as the effects may surpass epithelial repair only. Additional beneficial effects of EP4 agonism in COPD may include bronchoprotection (in humans; EP2 in mice) (*36*) and inhibition of inflammation (*37*). Expression of the EP4 receptor is maintained in COPD and in smokers. Smoking and aging are associated with increased expression of PGE_2_ in the human lung (*38*, *39*). Whereas this may serve as an endogenous protective mechanism, PGE_2_ has multiple effects, and not all of these are protective to the pathophysiology of COPD. We propose that selective EP4 agonism could unify several functional features that support the treatment of COPD, making this an intriguing pharmacological target.

Iloprost, a stable PGI_2_ analog (*30*, *40*–*44*), has been shown to have anti-inflammatory effects and protects against bleomycin-induced pulmonary fibrosis in mice (*41*) and is also clinically used for the treatment of pulmonary hypertension (*40*). Although a recent study (*43*) showed that iloprost improved clinical outcomes in patients with COPD with poor lung oxygenation, its impact on alveolar repair is unknown. Here, we show that iloprost prevents the repressed organoid formation resulting from CS(E) exposure. Whereas IP and EP4 receptors are both linked to cAMP generation, rolipram and olodaterol had no effect on organoid growth and only very limited effect when applied in combination. This apparent discrepancy might be explained by cAMP compartmentalization. This phenomenon explains why and how distinct G_s_ protein–coupled receptors or drugs activating the cAMP pathway can have distinct effects on cAMP generation and function (*45*). An alternative explanation is that EP4 receptor expression is higher than that of the β_2_-adrenergic receptor. As corticosteroids have been shown to inhibit the release of arachidonic acid metabolites including PGI_2_ (*46*) and PGE_2_ (*17*), we speculate that the negative effect of budesonide on the progenitor cell growth may be due to the restriction of PG synthesis.

Analogs of PGE_2_ and PGI_2_ have previously been demonstrated to attenuate other features of COPD in mice. For example, iloprost attenuated inflammation, airway hyperresponsiveness, and airway remodeling in a mouse model of lipopolysaccharide and elastase exposure (*45*). Systemically administered EP2 agonists have beneficial effects on angiogenesis in a mouse model of emphysema (*45*). Moreover, PGE_2_ represses fibroblast activation, fibroblast to myofibroblast differentiation, and migratory capacity (*47*). PGE_2_ has been reported (*48*) as a key regulator released from alveolar epithelial cells regulating the barrier function of the lung microvasculature. However, we did not see supportive effects of misoprostol or iloprost using a scratch wound assay on human pulmonary endothelial cells exposed to CS (fig. S8), indicating that the beneficial effects of PGE_2_ and PGI_2_ on repair may be specific for the alveolar epithelial cell. In addition, studies have shown PGE_2_ to be anti-inflammatory by attenuating macrophages activation and proliferation (*39*, *49*), While beneficial, the anti-inflammatory activity of PGE_2_ and PGI_2_ analogs could also predispose to pulmonary infections, which is a risk that needs to be considered moving forward. Here, we identified the potential therapeutic effect of PGE_2_ and PGI_2_ analogs on defective alveolar progenitors in response to CS that appears to be primarily related to the disturbance of circadian clock signaling rather than its anti-inflammatory response. Given the diverse and occasionally opposite roles of PGE_2_ and PGI_2_ in the pathophysiology of COPD, the main challenge will be to balance broad spectrum with specificity, for example, using subtype-selective drugs or drugs targeted to specific lung cell types.

By generating transcriptomic signatures of epithelial progenitors derived from mice exposed in vivo to air, CS, CS + misoprostol, or CS + iloprost, we uncovered dynamic molecular signaling pathways in response to CS exposure. We identified circadian clock signaling as being significantly repressed in the alveolar epithelial progenitors derived from mice exposed to CS, which could be improved by either misoprostol or iloprost treatment. Circadian rhythms (*50*–*53*) are normally generated and regulated by clock genes, including *BMAL1* (*ARNTL1*) and *CLOCK* encoding activators, period (*PER1* to *PER3*) and cryptochrome genes (*CRY1* and *CRY2*) that encode repressors, and the nuclear receptors Rev-erb (*NR1D1* and *NR1D2*) and *RORA*, which constitute secondary regulatory loops. These core clock genes not only activate or repress a cell-autonomous clock but also regulate the clock-controlled genes (*54*), thus interacting with other molecular signaling pathways. Previously, it has been demonstrated that clock signaling is down-regulated in CS-exposed mice, linked to an impairment of antioxidant defense mechanisms (*55*), and Rev-erbα has been shown as an key regulator of inflammatory response in lung injury models (*19*–*21*, *56*). Here, we show that the Rev-erbα agonist prevents the impaired organoid formation resulted from CSE exposure, suggesting that PGE_2_/PGI_2_/Rev-erbα signaling pathway plays an important role in defective lung repair.

Furthermore, we found that CS exposure up-regulated pathways associated with cell cycle activity in alveolar epithelial progenitors, which could be counteracted by in vivo misoprostol or iloprost treatment. The cell cycle (*57*–*61*) is driven by a set of tightly regulated molecular events controlling DNA replication and mitosis with four phases, and each individual cell may require different triggers to decide whether to enter proliferation or apoptosis. To further assess alveolar epithelial progenitors under which cell cycle/apoptotic status in response to CS exposure and additional PGE_2_/PGI_2_ treatments may be the next step to investigate in the future. A link between circadian clock and cell cycle signaling pathways has been proposed (*54*, *60*, *62*). The molecular control of the biological clock is dependent on cAMP signaling, and cAMP activators are known to entrain the biological clock (*63*), explaining the link between PGE_2_ and PGI_2_ activations and restoration of the defective clock signaling in combination with CS exposure. Hence, it is of great interest to determine in more molecular detail how these two oscillatory systems communicate in regulating PGE_2_/PGI_2_-mediated lung repair in future studies.

In conclusion, in this study, we demonstrate the protective effects of several drug candidates, including PGE_2_ and PGI_2_ analogs, against in vivo and in vitro CS(E)-induced damage of alveolar epithelial progenitors. Furthermore, using transcriptome analysis, we show that CS induces a wide range of transcriptional changes, including alterations of circadian clock and cell cycle signaling pathways, which can be counteracted by either misoprostol (PGE_2_) or iloprost (PGI_2_) treatment. While the focus of our studies was on COPD, similar beneficial effects may be operative in diseases affected by similar defects in alveolar progenitors such as acute respiratory distress syndrome and pulmonary fibrosis. Overall, these data provide promising therapeutic strategies to specifically address defective lung repair in respiratory diseases, in particular, targeting EP4 and IP receptors.

## MATERIALS AND METHODS

### Animals

Mouse experiments for organoid study were performed at the Central Animal Facility of the University Medical Center Groningen (UMCG) in accordance with the national guidelines and upon approval of the experimental procedures by CDP and the Institutional Animal Care and Use Committee of the University of Groningen (license AVD105002015303). C57BL/6J (555) and BALB/cByJ (Jax strain) mice (both genders, 8 to 12 weeks of age) were maintained under a 12-hour light/dark cycles and were allowed food and water ad libitum. Animals for circadian clock studies were exposed to CS and/or administrated with compounds at the same time of the day for all mice in all groups. Animals were euthanized at the same time of the day. Animals for the chronic CS model were adult (female, 8 to 10 weeks of age at the start of the study) C57BL/6N mice, which were obtained from the Charles River Laboratories (Sulzfeld, Germany) and used for both the scRNA-seq analysis and organoid assay. These experiments were performed at the Helmholtz Zentrum München and approved by the ethics committee for animal welfare of the local government for the administrative region of Upper Bavaria (Regierungspräsidium Oberbayern) and were conducted under strict governmental and international guidelines in accordance with European Union Directive 2010/63/EU.

### Human material

The human lung tissue was obtained from lung transplant donors according to the Eurotransplant guidelines including the absence of primary lung diseases such as asthma and COPD and no more than 20 pack years of smoking history (*64*). Human lung tissue specimens were obtained with full informed consent from patients undergoing lung volume reduction surgery or lung transplantation at UMCG. The study protocol was consistent with the Research Code of the UMCG and national ethical and professional guidelines. All tissue samples were anonymized before use. Gene expression in human lung published datasets was obtained by downloading a series matrix files from the National Center for Biotechnology Information (NCBI) Gene Expression Omnibus (GEO) database for GSE76925 (*65*) and gene expression normalized to healthy smokers.

### In vivo CS exposure

Mice (*n* = 4 to 11 per group, 10 to 12 weeks old) were exposed (whole body) to 3R4F research cigarettes (Tobacco Research Institute, University of Kentucky, Lexington, KY) for four consecutive days (two sessions per day, 8 hours between each exposure) to establish an acute smoke-induced inflammation model, as described previously (*9*). In the CS group, mice were exposed to one cigarette in the morning and three in the afternoon on day 1. From days 2 to 4, mice were exposed to five cigarettes each session. All cigarettes were smoked without a filter in 5 min at a rate of 5 liter/hour in a ratio with 60 liter/hour air using a peristaltic pump (45 rpm; Watson Marlow 323 E/D, Rotterdam, the Netherlands). In the control group, mice were exposed to fresh air using similar exposure chambers as the CS group.

In some studies, budesonide was nebulized (0.1 mM; 15 min per mouse per exposure) to wild-type mice (*n* = 6) before each CS exposure. In separate studies, intraperitoneal injections of 50 μg of misoprostol or 50 μg of iloprost were given to wild-type mice (*n* = 6 to 8) 30 min before each CS exposure. On day 5, mice were euthanized, and the lungs were immediately used for establishing organoid cultures or stored at −80°C for further experimental uses.

For the scRNA-seq analysis and the organoid assay setup in the chronic model, CS was generated from 3R4F research cigarettes, with the filters removed to expose to the murine lungs. Mice were whole body exposed to active 100% mainstream CS of 500 mg/m^3^ total particulate matter for 50 min twice per day for 4 m in a manner mimicking natural human smoking habits as previously described (*65*).

### Fibroblast culture

Mouse fibroblasts, CCL-206 [Mlg (CCL-206); American Type Culture Collection (ATCC), Wesel, Germany] were cultured in Dulbecco’s modified Eagle’s medium (DMEM)/F12 medium supplemented with 10% (v/v) fetal bovine serum (FBS), penicillin/streptomycin (100 U/ml), 2 mM l-glutamine, and 1% amphotericin B in a humidified atmosphere under 5% CO_2_/95% air at 37°C, as previously described (*6*, *10*, *66*). For organoid experiments, fibroblasts were proliferation-inactivated by incubation in mitomycin C (10 μg/ml; M4287, Sigma-Aldrich) for 2 hours, followed by three washes with phosphate-buffered saline (PBS), after which the cells were trypsinized before introduction into the organoid cocultures. Human lung fibroblasts MRC5 (CCL-171, ATCC, Wesel, Germany) were cultured in Ham’s F12 medium supplemented with the same additives as the murine fibroblasts’ medium.

### Cigarette smoke extract

The smoke of two 3R4F research cigarettes was pumped into 25 ml of warm fibroblasts culture medium to produce 100% CSE (*12*). All cigarettes were without a filter, and smoke was passed through the medium using a peristaltic pump (45 rpm; Watson Marlow 323 E/D, Rotterdam, the Netherlands). CSE was freshly prepared before each set of experiments.

### Murine primary alveolar epithelial isolation

The primary alveolar epithelial cells, brief in Epcam^+^ cells (CD31^−^/CD45^−^/CD326^+^) were isolated on the basis of the protocols published previously from our group (*67*). The isolation of Epcam^+^ cells generated from the chronic CS mice was slightly different. Murine lungs harvested from mice exposed with CS (4 months) were stored in MACS tissue storage solution (130-100-008) at 4°C overnight (due to shipment). Lungs were chopped into small pieces and incubated in the enzyme mix containing dispase (#354235, Corning) and deoxyribonuclease I (A3778, Analytics-Shop) for 1 hour at 37°C. The rest procedure is the same as the ordinary epithelial isolation.

### Organoid culture

The adult alveolar epithelial progenitor cell–derived organoid assay provides a platform to study the response of alveolar epithelial cells to various conditions rapidly and easily where complex in vivo analysis is less feasible (*68*) and typical two-dimensional cell culture cannot recapitulate the cell-cell interactions. Moreover, organoids represent a powerful tool to model various pathological states of environmental or genetic origin that can be easily manipulated in vitro for validation of therapeutic approaches (*69*). The organoid culture system is based on previously published protocols from our group (*6*, *10*, *66*). Briefly, epithelial cells (CD31^−^/CD45^−^/CD326^+^) were freshly isolated from murine or human lung tissue and cocultured with murine CCL-206 or human MRC5 fibroblasts, respectively, in Matrigel (Corning Life Sciences B.V., Amsterdam, The Netherlands). EpCAM^+^ (CD31^−^/CD45^−^/CD326^+^) cells were isolated from mouse lung tissue (without the trachea) using the QuadroMACS Separator and antibody-bound magnetic beads (Miltenyi Biotec, Leiden, The Netherlands). EpCAM^+^ cells and fibroblasts were mixed 1:1 (20,000 cells each) and suspended in 100 μl of Matrigel prediluted 1:1 (v/v) with DMEM supplemented with 10% FBS. This mixture of cells was added to a 24-well Falcon cell culture insert (Corning, USA) within a 24-well plate containing 400 μl of organoid media [DMEM/F-12 supplemented with 5% FBS, 1% penicillin/streptomycin, 1% glutamine, 1% amphotericin B, 0.025 per mil (‰) epidermal growth factor, 1% insulin-transferrin-selenium, and 1.75‰ bovine pituitary extract] underneath the insert in each well. Adult human donor tissue was isolated from histologically normal regions of lung tissue specimens obtained at UMCG (Groningen, The Netherlands) from *n* = 7 patients (two non-COPD and five patients with COPD). Human lung tissues were incubated and homogenized overnight in an enzyme mixture at 4°C; the EpCAM^+^ isolation process was similar to that described above for murine lung tissue. Organoids were cultured in a humidified atmosphere under 5% CO_2_/95% air at 37°C, and medium in the wells was refreshed every 2 to 3 days. To quantify the number of organoids, light microscopy at ×20 magnification was used, and organoids were counted manually. The diameter of the organoids (organoid size) was measured using NIS-Elements software with a light microscope. The number parameter represents the ability of alveolar epithelial progenitors to form organoids, whereas the size parameter may result from swelling or proliferation. Subsequent phenotyping identifies effects on alveolar type II cell differentiation.

For in vitro organoid experiments, organoids were continuously treated with control, 1.25 (1% for human organoids), 2.5, or 5% CSE; and organoid culture medium was refreshed every other day. All information about the pharmacological compounds used in this study is provided in the table S1.

### Immunofluorescence staining

The immunofluorescence staining assay for organoids was performed as described previously by our group with minor modifications (*6*, *10*, *66*). Organoids were fixed in acetone diluted 1:1 (v/v) with methanol for 15 min at −20°C. After fixation, 1 ml of PBS with 0.02% sodium azide was added to the well underneath the insert. Organoids were kept at 4°C for 1 week after fixation. Bovine serum albumin (BSA) medium was added on top of the insert for blocking at room temperature (RT) for 2 hours. Afterward, primary antibody incubation was performed in PBS buffer with 0.1% BSA and 0.1% Triton X-100 overnight at 4°C. The next day, the organoids were washed three times with PBS for 30 min, and secondary antibody incubation was performed for 2 hours at RT. After washing with PBS for 15 min, the organoids on the insert membrane were transferred to a glass slide with two drops of mounting medium containing 4 the organoids on the insert (DAPI) (104139, Abcam, Cambridge, UK), and a coverslip was applied.

The immunofluorescence staining assay for the lung slices was performed as described previously with minor modifications (*6*). The frozen murine lung sections (5 μm) were dried for 30 min and then were fixed in 4% paraformaldehyde/PBS solution at RT for 15 min. After rinsing three times with PBS, the lung sections were blocked within PBS buffer containing 5% donkey serum and 0.3% Triton X-100 at RT for 60 min. The primary antibody incubation [Rev-erbAα/NR1D1 antibody, NBP2-75645, Novus Biologicals (1:100 dilution); anti-PER3 antibody, ab224594, Abcam (1:100 dilution); and anti–pro-SPC antibody, AB3786, Sigma-Aldrich (1:100 dilution)] was performed in the PBS buffer containing 1% BSA and 0.3% Triton X-100 at 4°C overnight. After rinsing three times with PBS, the lung sections were incubated with secondary antibodies (donkey anti-rabbit immunoglobulin G Alexa Fluor 488, A21206, Thermo Fisher Scientific; 1:1000 dilution) at RT for 2 hours. The sections were rinsed twice with PBS and once with ultrapure water and then were transferred to a glass slide, and then the coverslip was applied.

The slides were kept at 4°C. Confocal images were acquired using a Leica SP8 microscope or a Leica DM4000B microscope. Images were obtained and analyzed with LAS X (Leica) software (open resource, Leica Microsystems GmbH, Wetzlar, Germany).

### RNA extraction and RNA-seq analysis

The Epcam^+^ cells isolated from mice exposed to air, CS, CS + misoprostol, or CS + iloprost were used to extract total RNA for RNA-seq using NucleoSpin RNA kit (740955, Macherey-Nagel, Germany) according to the manufacturer’s instructions. RNA concentrations and qualities were analyzed using NanoDrop spectrophotometer. An Illumina NovaSeq 6000 sequencer was used for the RNA-seq data analysis by GenomeScan (www.genomescan.nl). The procedure included data quality control, adapter trimming, alignment of short reads, and feature counting. Library preparation was checked by calculating the ribosomal (and globin) content. Checks for possible sample and barcode contamination were performed, and a set of standard quality metrics for the raw dataset was determined using quality control tools (FastQC v0.34 and FastQA). Before alignment, the reads were trimmed for adapter sequences using Trimmomatic v0.30. To align the reads of each sample, the ensemble mouse reference GRCm38 (patch 6) was used. Analyses following the RNA-seq studies were performed using the BioJupies platform (https://amp.pharm.mssm.edu/biojupies/) (*70*) and using the DeSeq2 package in R. Gene expression in murine lung published datasets was obtained by downloading a series of matrix files from the NCBI GEO database (GSE151674).

### Immunohistochemistry

Immunohistochemistry was performed on the murine lung tissue harvested from mice exposed by air/CS/CS + misoprostol (intraperitoneal)/CS + iloprost (intraperitoneal) based on the optimized protocol described previously (*71*). Briefly, paraffin-embedded frozen murine lung tissue was cut with a Microm HM 340E microtome. Five-micrometer transverse cross sections were used for analysis. Tissue sections were first dried 30 min with hair blower and then were rehydrated in PBS. Tissue sections were stained with 3,3′-diamino-benzidine (DAB) (Sigma-Aldrich) NaCN and H_2_O_2_ for 7 min. After rising within PBS, sections were counterstained with hematoxylin and imbedded in Kaiser gelatin. Sections were stained with Mayer’s hematoxylin for 10 min, rinsed in running tap water for 10 min, and then were stained with eosin for 3 min. After a short rinsing step (UP water), the sections were quickly dehydrated in 96% ethanol and 100% ethanol and xylene. The section was mounted with mounting medium.

### Statistics analysis

All data are presented as means ± SEM unless indicated otherwise. Unless stated otherwise, all data were assessed for statistical significance using two-tailed Student’s *t* test or one-way analysis of variance (ANOVA). The *P* value indicating statistically significant differences between the mean/median values are defined as follows: *P < 0.05, **P < 0.01, ***P < 0.001, and ****P < 0.0001. Statistical analyses were performed with GraphPad Prism 8 software.

## Supplementary Materials

### Other Supplementary Material for this manuscript includes the following:

Table S3

Datasets S1 to S3

## References

[1] N. Kokturk, F. Yıldırım, P. Y. Gülhan, Y. M. Oh, Stem cell therapy in chronic obstructive pulmonary disease. How far is it to the clinic? Am. J. Stem Cells. 7, 56–71 (2018). 30245915 PMC6146161

[2] M. C. Basil, J. Katzen, A. E. Engler, M. Guo, M. J. Herriges, J. J. Kathiriya, R. Windmueller, A. B. Ysasi, W. J. Zacharias, H. A. Chapman, D. N. Kotton, J. R. Rock, H. W. Snoeck, G. Vunjak-Novakovic, J. A. Whitsett, E. E. Morrisey, The cellular and physiological basis for lung repair and regeneration: Past, present, and future. Cell Stem Cell 26, 482–502 (2020). 32243808 10.1016/j.stem.2020.03.009 PMC7128675

[3] T. Volckaert, T. Yuan, C. M. Chao, H. Bell, A. Sitaula, L. Szimmtenings, E. El Agha, D. Chanda, S. Majka, S. Bellusci, V. J. Thannickal, R. Fässler, S. P. De Langhe, Fgf10-hippo epithelial-mesenchymal crosstalk maintains and recruits lung basal stem cells. Dev. Cell 43, 48–59.e5 (2017). 29017029 10.1016/j.devcel.2017.09.003 PMC5679744

[4] Y. Hu, J. P. Ng-Blichfeldt, C. Ota, C. Ciminieri, W. Ren, P. S. Hiemstra, J. Stolk, R. Gosens, M. Königshoff, Wnt/β-catenin signaling is critical for regenerative potential of distal lung epithelial progenitor cells in homeostasis and emphysema. Stem Cells 38, 1467–1478 (2020). 32526076 10.1002/stem.3241 PMC7116441

[5] T. M. Conlon, G. John-Schuster, D. Heide, D. Pfister, M. Lehmann, Y. Hu, Z. Ertüz, M. A. Lopez, M. Ansari, M. Strunz, C. Mayr, C. Ciminieri, R. Costa, M. S. Kohlhepp, A. Guillot, G. Günes, A. Jeridi, M. C. Funk, G. Beroshvili, S. Prokosch, J. Hetzer, S. E. Verleden, H. Alsafadi, M. Lindner, G. Burgstaller, L. Becker, M. Irmler, M. Dudek, J. Janzen, E. Goffin, R. Gosens, P. Knolle, B. Pirotte, T. Stoeger, J. Beckers, D. Wagner, I. Singh, F. J. Theis, M. H. de Angelis, T. O’Connor, F. Tacke, M. Boutros, E. Dejardin, O. Eickelberg, H. B. Schiller, M. Königshoff, M. Heikenwalder, A. Ö. Yildirim, Inhibition of LTβR signalling activates WNT-induced regeneration in lung. Nature 588, 151–156 (2020). 33149305 10.1038/s41586-020-2882-8 PMC7718297

[6] X. Wu, E. M. van Dijk, J.-P. Ng-Blichfeldt, I. S. T. Bos, C. Ciminieri, M. Königshoff, L. E. M. Kistemaker, R. Gosens, Mesenchymal WNT-5A/5B signaling represses lung alveolar epithelial progenitors. Cells 8, 1147 (2019). 31557955 10.3390/cells8101147 PMC6829372

[7] E. Samaha, K. Vierlinger, W. Weinhappel, J. Godnic-Cvar, C. Nöhammer, D. Koczan, H. J. Thiesen, H. Yanai, V. E. Fraifeld, R. Ziesche, Expression profiling suggests loss of surface integrity and failure of regenerative repair as major driving forces for chronic obstructive pulmonary disease progression. Am. J. Respir. Cell Mol. Biol. 64, 441–452 (2021). 33524306 10.1165/rcmb.2020-0270OC

[8] C. A. Brandsma, M. Van Den Berge, D. S. Postma, M. R. Jonker, S. Brouwer, P. D. Paré, D. D. Sin, Y. Bossé, M. Laviolette, J. Karjalainen, R. S. N. Fehrmann, D. C. Nickle, K. Hao, A. I. R. Spanjer, W. Timens, L. Franke, A large lung gene expression study identifying fibulin-5 as a novel player in tissue repair in COPD. Thorax 70, 21–32 (2015). 24990664 10.1136/thoraxjnl-2014-205091

[9] L. E. M. Kistemaker, R. P. Van Os, A. Dethmers-Ausema, I. Sophie, M. N. Hylkema, M. Van Den Berge, P. S. Hiemstra, J. Wess, H. Meurs, H. A. M. Kerstjens, R. Gosens, Muscarinic M3 receptors on structural cells regulate cigarette smoke-induced neutrophilic airway inflammation in mice. Am. J. Physiol. Lung Cell. Mol. Physiol. 308, L96–L103 (2015). 25381025 10.1152/ajplung.00259.2014 PMC4315453

[10] J. P. Ng-Blichfeldt, A. Schrik, R. K. Kortekaas, J. A. Noordhoek, I. H. Heijink, P. S. Hiemstra, J. Stolk, M. Königshoff, R. Gosens, Retinoic acid signaling balances adult distal lung epithelial progenitor cell growth and differentiation. EBioMedicine 36, 461–474 (2018). 30236449 10.1016/j.ebiom.2018.09.002 PMC6197151

[11] B. Woodby, C. Sticozzi, E. Pambianchi, G. Villetti, M. Civelli, G. Valacchi, F. Facchinetti, The PDE4 inhibitor CHF6001 affects keratinocyte proliferation via cellular redox pathways. Arch. Biochem. Biophys. 685, 108355 (2020). 32268137 10.1016/j.abb.2020.108355

[12] H. Zuo, B. Han, W. J. Poppinga, L. Ringnalda, L. E. M. Kistemaker, A. J. Halayko, R. Gosens, V. O. Nikolaev, M. Schmidt, Cigarette smoke up-regulates PDE3 and PDE4 to decrease cAMP in airway cells. Br. J. Pharmacol. 175, 2988–3006 (2018). 29722436 10.1111/bph.14347 PMC6016635

[13] T. B. Kothe, F. H. Sadiq, N. Burleyson, H. L. Williams, C. Anderson, N. H. Hillman, Surfactant and budesonide for respiratory distress syndrome: An observational study. Pediatr. Res. 87, 940–945 (2020). 31715622 10.1038/s41390-019-0663-6

[14] F. Ricci, C. Catozzi, F. Ravanetti, X. Murgia, F. D’Aló, N. Macchidani, E. Sgarbi, V. Di Lallo, F. Saccani, M. Pertile, A. Cacchioli, S. Catinella, G. Villetti, M. Civelli, F. Amadei, F. F. Stellari, B. Pioselli, F. Salomone, In vitro and in vivo characterization of poractant alfa supplemented with budesonide for safe and effective intratracheal administration. Pediatr. Res. 82, 1056–1063 (2017). 28723887 10.1038/pr.2017.171

[15] D. P. Tashkin, B. Lipworth, R. Brattsand, Benefit: Risk profile of budesonide in obstructive airways disease. Drugs 79, 1757–1775 (2019). 31549299 10.1007/s40265-019-01198-7 PMC6825643

[16] R. C. Waters, G. Hochhaus, Characterization of a dextran-budesonide prodrug for inhalation therapy. Eur. J. Pharm. Sci. 129, 58–67 (2019). 30521945 10.1016/j.ejps.2018.11.038

[17] S. M. Wilson, P. Shen, C. F. Rider, S. L. Traves, D. Proud, R. Newton, M. A. Giembycz, Selective prostacyclin receptor agonism augments glucocorticoid-induced gene expression in human bronchial epithelial cells. J. Immunol. 183, 6788–6799 (2009). 19880449 10.4049/jimmunol.0902738

[18] M. S. B. Raredon, T. S. Adams, Y. Suhail, J. C. Schupp, S. Poli, N. Neumark, K. L. Leiby, A. M. Greaney, Y. Yuan, C. Horien, G. Linderman, A. J. Engler, D. J. Boffa, Y. Kluger, I. O. Rosas, A. Levchenko, N. Kaminski, L. E. Niklason, Single-cell connectomic analysis of adult mammalian lungs. Sci. Adv. 5, eaaw3851 (2019). 31840053 10.1126/sciadv.aaw3851 PMC6892628

[19] W. Shen, W. Zhang, W. Ye, H. Wang, Q. Zhang, J. Shen, Q. Hong, X. Li, G. Wen, T. Wei, J. Zhang, SR9009 induces a REV-ERB dependent anti-small-cell lung cancer effect through inhibition of autophagy. Theranostics 10, 4466–4480 (2020). 32292508 10.7150/thno.42478 PMC7150483

[20] H. J. Durrington, K. Krakowiak, P. Meijer, N. Begley, R. Maidstone, L. Goosey, J. E. Gibbs, J. F. Blaikley, L. G. Gregory, C. M. Lloyd, A. S. I. Loudon, D. W. Ray, Circadian asthma airway responses are gated by REV-ERBα. Eur. Respir. J. 56, 1902407 (2020). 32586876 10.1183/13993003.02407-2019 PMC7613655

[21] I. K. Sundar, K. Rashid, M. T. Sellix, I. Rahman, The nuclear receptor and clock gene REV-ERBα regulates cigarette smoke-induced lung inflammation. Biochem. Biophys. Res. Commun. 493, 1390–1395 (2017). 28974420 10.1016/j.bbrc.2017.09.157 PMC5756581

[22] J. A. Gindele, T. Kiechle, K. Benediktus, G. Birk, M. Brendel, F. Heinemann, C. T. Wohnhaas, M. LeBlanc, H. Zhang, Y. Strulovici-Barel, R. G. Crystal, M. J. Thomas, B. Stierstorfer, K. Quast, J. Schymeinsky, Intermittent exposure to whole cigarette smoke alters the differentiation of primary small airway epithelial cells in the air-liquid interface culture. Sci. Rep. 10, 6257 (2020). 32277131 10.1038/s41598-020-63345-5 PMC7148343

[23] R. Munir, J. Lisec, J. V. Swinnen, N. Zaidi, Lipid metabolism in cancer cells under metabolic stress. Br. J. Cancer 120, 1090–1098 (2019). 31092908 10.1038/s41416-019-0451-4 PMC6738079

[24] X. Gao, S. H. Lin, F. Ren, J. T. Li, J. J. Chen, C. B. Yao, H. Bin Yang, S. X. Jiang, G. Q. Yan, D. Wang, Y. Wang, Y. Liu, Z. Cai, Y. Y. Xu, J. Chen, W. Yu, P. Y. Yang, Q. Y. Lei, Acetate functions as an epigenetic metabolite to promote lipid synthesis under hypoxia. Nat. Commun. 7, 11960 (2016). 27357947 10.1038/ncomms11960 PMC4931325

[25] M. P. Whyte, S. D. Amalnath, W. H. McAlister, M. D. McKee, D. J. Veis, M. Huskey, S. Duan, V. N. Bijanki, S. Alur, S. Mumm, Hypophosphatemic osteosclerosis, hyperostosis, and enthesopathy associated with novel homozygous mutations of DMP1 encoding dentin matrix protein 1 and SPP1 encoding osteopontin: The first digenic SIBLING protein osteopathy? Bone 132, 115190 (2020). 31843680 10.1016/j.bone.2019.115190 PMC7271119

[26] S. Shintani, N. Kamakura, M. Kobata, S. Toyosawa, T. Onishi, A. Sato, K. Kawasaki, K. M. Weiss, T. Ooshima, Identification and characterization of integrin-binding sialoprotein (IBSP) genes in reptile and amphibian. Gene 424, 11–17 (2008). 18723083 10.1016/j.gene.2008.07.035

[27] L. Zhang, X. Hou, S. Lu, H. Rao, J. Hou, R. Luo, H. Huang, H. Zhao, H. Jian, Z. Chen, M. Liao, X. Wang, Predictive significance of bone sialoprotein and osteopontin for bone metastases in resected Chinese non-small-cell lung cancer patients: A large cohort retrospective study. Lung Cancer 67, 114–119 (2010). 19376608 10.1016/j.lungcan.2009.03.017

[28] X. Guo, M. R. Roberts, S. M. Becker, B. Podd, Y. Zhang, S. C. Chua, M. G. Myers, P. Duggal, E. R. Houpt, W. A. Petri, Leptin signaling in intestinal epithelium mediates resistance to enteric infection by *Entamoeba histolytica*. Mucosal Immunol. 4, 294–303 (2011). 21124310 10.1038/mi.2010.76 PMC3079783

[29] N. J. N. N. P. S. Daniel, E. Shumer, Leptin receptor polymorphisms and lung function decline in COPD. Physiol. Behav. 176, 139–148 (2017).28363838

[30] K. Lee, S. H. Lee, T. H. Kim, The biology of prostaglandins and their role as a target for allergic airway disease therapy. Int. J. Mol. Sci. 21, 1851 (2020). 32182661 10.3390/ijms21051851 PMC7084947

[31] Y. Take, S. Koizumi, A. Nagahisa, Prostaglandin E receptor 4 antagonist in cancer immunotherapy: Mechanisms of action. Front. Immunol. 11, 1851 (2020). 32210957 10.3389/fimmu.2020.00324 PMC7076081

[32] I. Dey, M. Lejeune, K. Chadee, Prostaglandin E2 receptor distribution and function in the gastrointestinal tract. Br. J. Pharmacol. 149, 611–623 (2006). 17016496 10.1038/sj.bjp.0706923 PMC2014644

[33] T. Markovič, Ž. Jakopin, M. S. Dolenc, I. Mlinarič-Raščan, Structural features of subtype-selective EP receptor modulators. Drug Discov. Today 22, 57–71 (2017). 27506873 10.1016/j.drudis.2016.08.003

[34] F. J. Nunez, N. A. Schulte, D. M. Fogel, J. Michalski, S. I. Rennard, R. B. Penn, M. L. Toews, R. S. Ostrom, Agonist-specific desensitization of PGE2-stimulated cAMP signaling due to upregulated phosphodiesterase expression in human lung fibroblasts. Naunyn Schmiedebergs Arch. Pharmacol. 393, 843–856 (2020). 31884570 10.1007/s00210-019-01800-5 PMC7328663

[35] S. L. Tilley, J. M. Hartney, C. J. Erikson, C. Jania, M. Nguyen, J. Stock, J. McNeisch, C. Valancius, R. A. Panettieri, R. B. Penn, B. H. Koller, Receptors and pathways mediating the effects of prostaglandin E_2_ on airway tone. Am. J. Physiol. Lung Cell. Mol. Physiol. 284, 599–606 (2003). 10.1152/ajplung.00324.2002 12618422

[36] J. Buckley, M. A. Birrell, S. A. Maher, A. T. Nials, D. L. Clarke, M. G. Belvisi, EP4 receptor as a new target for bronchodilator therapy. Thorax 66, 1029–1035 (2011). 21606476 10.1136/thx.2010.158568 PMC3221321

[37] M. A. Birrell, S. A. Maher, B. Dekkak, V. Jones, S. Wong, P. Brook, M. G. Belvisi, Anti-inflammatory effects of PGE2 in the lung: Role of the EP4 receptor subtype. Thorax 70, 740–747 (2015). 25939749 10.1136/thoraxjnl-2014-206592 PMC4516010

[38] M. Profita, A. Sala, A. Bonanno, L. Riccobono, M. Ferraro, S. La Grutta, G. D. Albano, A. M. Montalbano, M. Gjomarkaj, Chronic obstructive pulmonary disease and neutrophil infiltration: Role of cigarette smoke and cyclooxygenase products. Am. J. Physiol. Lung Cell. Mol. Physiol. 298, L261–L269 (2010). 19897740 10.1152/ajplung.90593.2008

[39] L. R. Penke, J. M. Speth, C. Draijer, Z. Zaslona, J. Chen, P. Mancuso, C. M. Freeman, J. L. Curtis, D. R. Goldstein, M. Peters-Golden, PGE_2_ accounts for bidirectional changes in alveolar macrophage self-renewal with aging and smoking. Life Sci. Alliance. 3, e202000800 (2020). 32820026 10.26508/lsa.202000800 PMC7441521

[40] L. Wang, Y. Z. Jin, Q. H. Zhao, R. Jiang, W. H. Wu, S. G. Gong, J. He, J. M. Liu, Z. C. Jing, Hemodynamic and gas exchange effects of inhaled iloprost in patients with COPD and pulmonary hypertension. Int. J. COPD. Volume 12, 3353–3360 (2017). 10.2147/COPD.S141798 PMC5702173 29200842

[41] Y. Zhu, Y. Liu, W. Zhou, R. Xiang, L. Jiang, K. Huang, Y. Xiao, Z. Guo, J. Gao, A prostacyclin analogue, iloprost, protects from bleomycin-induced pulmonary fibrosis in mice. Respir. Res. 11, 34 (2010). 20302663 10.1186/1465-9921-11-34 PMC2848635

[42] B. Gohrbandt, S. P. Sommer, S. Fischer, J. M. Hohlfeld, G. Warnecke, A. Haverich, M. Strueber, Iloprost to improve surfactant function in porcine pulmonary grafts stored for twenty-four hours in low-potassium dextran solution. J. Thorac. Cardiovasc. Surg. 129, 80–86 (2005). 15632828 10.1016/j.jtcvs.2004.04.040

[43] N. Kim, S. H. Lee, Y. Joe, T. Kim, H. Shin, Y. J. Oh, Effects of inhaled iloprost on lung mechanics and myocardial function during one-lung ventilation in chronic obstructive pulmonary disease patients combined with poor lung oxygenation. Anesth. Analg. 130, 1407–1414 (2020). 32167976 10.1213/ANE.0000000000004733

[44] S. H. Lee, J. G. Lee, C. Y. Lee, N. Kim, M. Y. Chang, Y. C. You, H. J. Kim, H. C. Paik, Y. J. Oh, Effects of intraoperative inhaled iloprost on primary graft dysfunction after lung transplantation: A retrospective single center study. Medicine 95, e3975 (2016). 27399072 10.1097/MD.0000000000003975 PMC5058801

[45] M. Schmidt, I. Cattani-Cavalieri, F. J. Nuñez, R. S. Ostrom, Phosphodiesterase isoforms and cAMP compartments in the development of new therapies for obstructive pulmonary diseases. Curr. Opin. Pharmacol. 51, 34–42 (2020). 32622335 10.1016/j.coph.2020.05.002 PMC7529846

[46] Z. J. Wang, D. J. Wilkie, R. Molokie, Neurobiological mechanisms of pain in sickle cell disease. Hematology 2010, 403–408 (2010). 21239826 10.1182/asheducation-2010.1.403 PMC3650026

[47] E. Elwakeel, B. Brüne, A. Weigert, PGE_2_ in fibrosis and cancer: Insights into fibroblast activation. Prostaglandins Other Lipid Mediat. 143, 106339 (2019). 31100473 10.1016/j.prostaglandins.2019.106339

[48] T. Bärnthaler, J. Maric, W. Platzer, V. Konya, A. Theiler, C. Hasenöhrl, B. Gottschalk, S. Trautmann, Y. Schreiber, W. F. Graier, R. Schicho, G. Marsche, A. Olschewski, D. Thomas, R. Schuligoi, A. Heinemann, The role of PGE2 in alveolar epithelial and lung microvascular endothelial crosstalk. Sci. Rep. 7, 7923 (2017). 28801643 10.1038/s41598-017-08228-y PMC5554158

[49] L. S. Saleh, C. Vanderheyden, A. Frederickson, S. J. Bryant, Prostaglandin E_2_ and its receptor EP_2_ modulate macrophage activation and fusion in vitro. ACS Biomater Sci. Eng. 6, 2668–2681 (2020). 33463295 10.1021/acsbiomaterials.9b01180

[50] A. Gallardo, A. Molina, H. G. Asenjo, J. Martorell-Marugán, R. Montes, V. Ramos-Mejia, A. Sanchez-Pozo, P. Carmona-Sáez, L. Lopez-Onieva, D. Landeira, The molecular clock protein Bmal1 regulates cell differentiation in mouse embryonic stem cells. Life Sci. alliance. 3, e201900535 (2020). 32284355 10.26508/lsa.201900535 PMC7156284

[51] S. Almeida, M. Chaves, F. Delaunay, Transcription-based circadian mechanism controls the duration of molecular clock states in response to signaling inputs. J. Theor. Biol. 484, 110015 (2020). 31539528 10.1016/j.jtbi.2019.110015

[52] A. Braghiroli, F. Braido, A. Piraino, P. Rogliani, P. Santus, N. Scichilone, Day and night control of copd and role of pharmacotherapy: A review. Int. J. COPD. 15, 1269–1285 (2020). 10.2147/COPD.S240033 PMC7283230 32606638

[53] X. Zou, D. W. Kim, T. Gotoh, J. Liu, J. K. Kim, C. V. Finkielstein, A systems biology approach identifies hidden regulatory connections between the circadian and cell-cycle checkpoints. Front. Physiol. 11, 1–9 (2020). 32372973 10.3389/fphys.2020.00327 PMC7176909

[54] J. Gaucher, E. Montellier, P. Sassone-Corsi, Molecular Cogs: Interplay between circadian clock and cell cycle. Trends Cell Biol. 28, 368–379 (2018). 29471986 10.1016/j.tcb.2018.01.006

[55] I. Rahman, Antioxidant therapies in COPD. Int. J. Chron. Obstruct. Pulmon. Dis. 1, 15–29 (2006). 18046899 10.2147/copd.2006.1.1.15 PMC2706605

[56] D. Yu, X. Fang, Y. Xu, H. Xiao, T. Huang, Y. Zhang, Y. Ge, Y. Li, L. Zong, J. Gao, Rev-erbα can regulate the NF-κB/NALP3 pathway to modulate lipopolysaccharide-induced acute lung injury and inflammation. Int. Immunopharmacol. 73, 312–320 (2019). 31129418 10.1016/j.intimp.2019.04.035

[57] K. G. Wiman, B. Zhivotovsky, Understanding cell cycle and cell death regulation provides novel weapons against human diseases. J. Intern. Med. 281, 483–495 (2017). 28374555 10.1111/joim.12609

[58] S. Maddika, S. R. Ande, S. Panigrahi, T. Paranjothy, K. Weglarczyk, A. Zuse, M. Eshraghi, K. D. Manda, E. Wiechec, M. Los, Cell survival, cell death and cell cycle pathways are interconnected: Implications for cancer therapy. Drug Resist. Updat. 10, 13–29 (2007). 17303468 10.1016/j.drup.2007.01.003

[59] J. Padgett, S. D. M. Santos, From clocks to dominoes: Lessons on cell cycle remodelling from embryonic stem cells. FEBS Lett. 594, 2031–2045 (2020). 10.1002/1873-3468.13862 32535913

[60] E. Farshadi, G. T. J. van der Horst, I. Chaves, Molecular links between the circadian clock and the cell cycle. J. Mol. Biol. 3515–3524 (2020). 32304699 10.1016/j.jmb.2020.04.003

[61] J. A. Zepp, E. E. Morrisey, Cellular crosstalk in the development and regeneration of the respiratory system. Nat. Rev. Mol. Cell Biol. 20, 551–566 (2019). 31217577 10.1038/s41580-019-0141-3 PMC7254499

[62] S. Almeida, M. Chaves, F. Delaunay, Control of synchronization ratios in clock/cell cycle coupling by growth factors and glucocorticoids. R. Soc. Open Sci. 7, 192054 (2020). 32257354 10.1098/rsos.192054 PMC7062057

[63] U. Abraham, A. E. Granada, P. O. Westermark, M. Heine, A. Kramer, H. Herzel, Coupling governs entrainment range of circadian clocks. Mol. Syst. Biol. 6, 438 (2010). 21119632 10.1038/msb.2010.92 PMC3010105

[64] L. E. M. Kistemaker, P. S. Hiemstra, I. S. T. Bos, S. Bouwman, M. Van Den Berge, M. N. Hylkema, H. Meurs, H. A. M. Kerstjens, R. Gosens, Tiotropium attenuates IL-13-induced goblet cell metaplasia of human airway epithelial cells. Thorax 70, 668–676 (2015). 25995156 10.1136/thoraxjnl-2014-205731

[65] J. D. Morrow, X. Zhou, T. Lao, Z. Jiang, D. L. Demeo, M. H. Cho, W. Qiu, S. Cloonan, V. Pinto-Plata, B. Celli, N. Marchetti, G. J. Criner, R. Bueno, G. R. Washko, K. Glass, J. Quackenbush, A. M. K. Choi, E. K. Silverman, C. P. Hersh, Functional interactors of three genome-wide association study genes are differentially expressed in severe chronic obstructive pulmonary disease lung tissue. Sci. Rep. 7, 44232 (2017). 28287180 10.1038/srep44232 PMC5347019

[66] J. P. Ng-Blichfeldt, T. de Jong, R. K. Kortekaas, X. Wu, M. Lindner, V. Guryev, P. S. Hiemstra, J. Stolk, M. Königshoff, R. Gosens, Tgf-β activation impairs fibroblast ability to support adult lung epithelial progenitor cell organoid formation. Am. J. Physiol. Lung Cell. Mol. Physiol. 317, L14–L28 (2019). 30969812 10.1152/ajplung.00400.2018

[67] X. Wu, V. Verschut, M. E. Woest, J. P. Ng-Blichfeldt, A. Matias, G. Villetti, A. Accetta, F. Facchinetti, R. Gosens, L. E. M. Kistemaker, Rho-kinase 1/2 inhibition prevents transforming growth factor-β-induced effects on pulmonary remodeling and repair. Front. Pharmacol. 11, 609509 (2021). 33551810 10.3389/fphar.2020.609509 PMC7855981

[68] M. C. Basil, E. E. Morrisey, Lung regeneration: A tale of mice and men. Semin. Cell Dev. Biol. 100, 88–100 (2020). 31761445 10.1016/j.semcdb.2019.11.006 PMC7909713

[69] M. Paschini, C. F. Kim, An airway organoid is forever. EMBO J. 38, e101526 (2019). 30718273 10.15252/embj.2019101526 PMC6376260

[70] D. Torre, A. Lachmann, A. Ma’ayan, BioJupies: Automated generation of interactive notebooks for RNA-Seq data analysis in the cloud. Cell Syst. 7, 556–561.e3 (2018). 30447998 10.1016/j.cels.2018.10.007 PMC6265050

[71] T. Koopmans, L. Hesse, M. C. Nawijn, K. Kumawat, M. H. Menzen, I. Sophie, R. Smits, E. R. M. Bakker, M. van den Berge, G. H. Koppelman, V. Guryev, R. Gosens, Smooth-muscle-derived WNT5A augments allergen-induced airway remodelling and Th2 type inflammation. Sci. Rep. 10, 6754 (2020). 32317758 10.1038/s41598-020-63741-x PMC7174298

