# Supplementary material for: A transcriptomics-guided drug target discovery strategy identifies receptor ligands for lung regeneration
Source: Sci Adv. 2022 Mar 23;8(12):eabj9949. doi: 10.1126/sciadv.abj9949 (PMC8942365; doi:10.1126/sciadv.abj9949)
Supplement: Supplementary file 1 — Figs. S1 to S8 Tables S1 and S2 [file sciadv.abj9949_sm.pdf]

Science Advances
AAAS

Supplementary Materials for

A transcriptomics-guided drug target discovery strategy identifies novel receptor ligands for lung regeneration

Xinhui Wu, I. Sophie T. Bos, Thomas M. Conlon, Meshal Ansari, Vicky Verschut, Luke van der Koog, Lars A. Verkleij, Angela D'Ambrosi, Aleksey Matveyenko, Herbert B. Schiller, Melanie Königshoff, Martina Schmidt, Loes E. M. Kistemaker, Ali Önder Yildirim, Reinoud Gosens*

*Corresponding author. Email: r.gosens@rug.nl

Published 23 March 2022, *Sci. Adv.* **8**, eabj9949 (2022)
DOI: 10.1126/sciadv.abj9949

The PDF file includes:

Figs. S1 to S8
Tables S1 to S2
Legend for table S3
Legends for datasets S1 to S3

Other Supplementary Material for this manuscript includes the following:

Table S3
Datasets S1 to S3

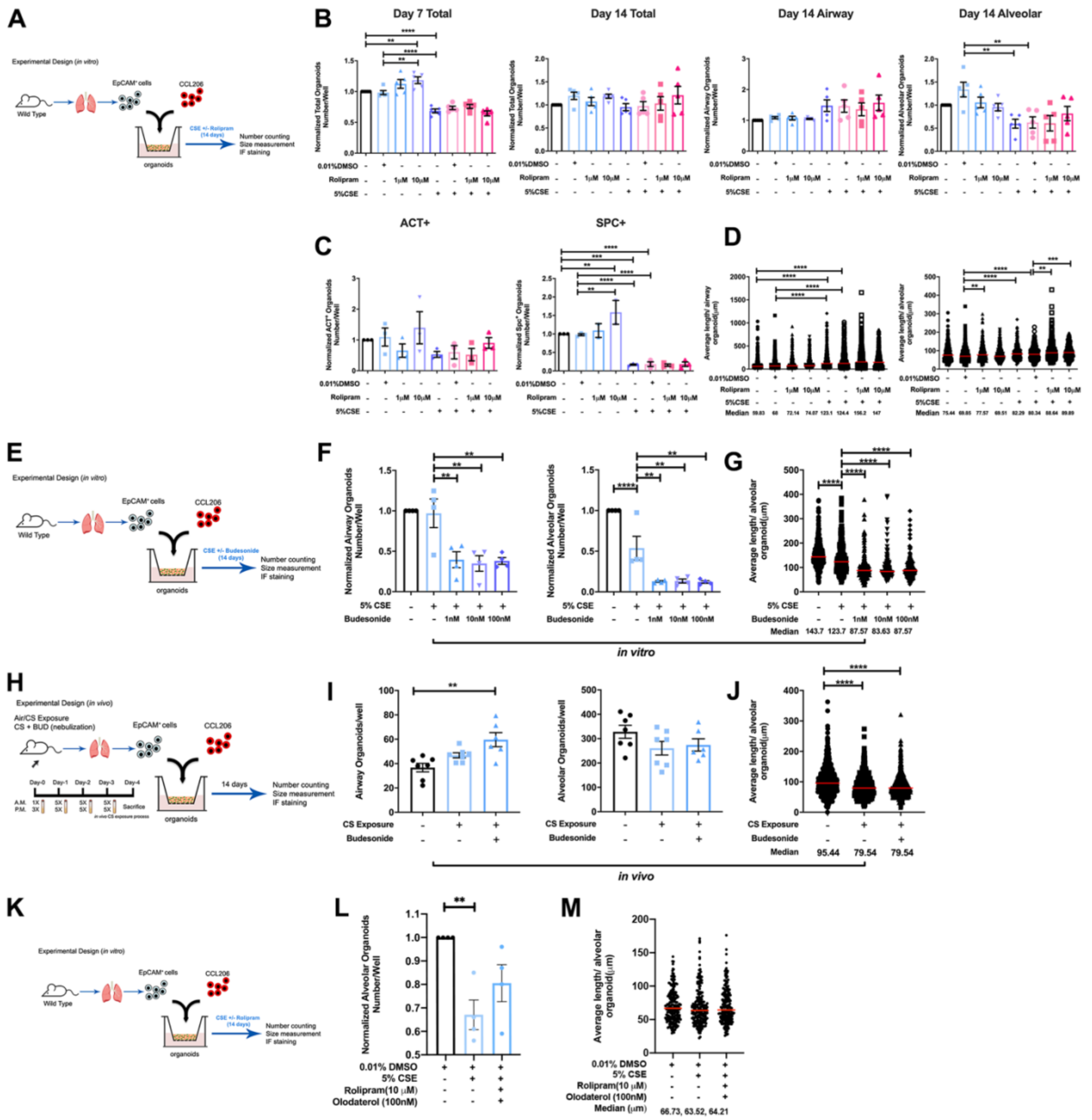

Fig. S1. Effect of Rolipram and Budesonide on lung organoid formation.

(**A**) Schematic of *in vitro* experimental design. (**B**) Quantification of normalized number of total organoids (day 7), total organoids (day 14), airway type organoids (day 14), alveolar type organoids (day 14) treated with 5% CSE ± rolipram (0-, 1-, 10μM). (**C**) Quantification of normalized ACT^{+} and SPC^{+} organoid numbers treated with 5% CSE ± rolipram (0-, 1-, 10μM) at Day 14. (**D**) Quantification of average length (diameter) of airway and alveolar type organoids treated with 5% CSE ± rolipram (0-, 1-, 10μM) measured on day 14. N = 5 experiments, n > 503 organoids/group. Data are presented as scatter plots with medians. (**E**) Schematic of *in vitro* experimental

design. (**F**) Quantification of normalized number of airway and alveolar type organoids treated with 5% CSE ± Budesonide (0-, 1-, 10-, 100 nM) measured on day 14. (**G**) Quantification of average length (diameter) of alveolar type organoids (median value) treated with 5% CSE ± Budesonide (0-, 1-, 10-, 100 nM) measured on day 14. N = 4 experiments, n > 165 organoids/group. Data are presented as scatter plots with medians. (**H**) Schematic of *in vivo* experimental design. (**I**) Number of airway and alveolar type organoids from co-culture of CCL-206 fibroblasts and $Epcam^{+}$ cells (isolated from air-exposed, CS-exposed, and CS-exposed + Budesonide nebulized mice) quantified on day 14. (**J**) Quantification of average length (diameter) of alveolar organoids (median value) from co-culture of CCL-206 fibroblasts and $Epcam^{+}$ cells (isolated from air-exposed, CS-exposed, and CS-exposed + Budesonide nebulized mice) on day 14. N = 6 - 7 experiments, n > 671 organoids/group. (**K**) Schematic of *in vitro* experimental design. (**L**) Quantification of normalized number of alveolar type organoids treated with 5% CSE ± Rolipram (10μM) + olodaterol (100 nM) measured on day 14. (**M**) Quantification of average length (diameter) of alveolar type organoids (median value) treated with 5% CSE ± Rolipram (10μM) + olodaterol (100 nM) measured on day 14. Data are presented as scatter plots with medians. **p < 0.05, **p < 0.01, ***p < 0.001, ****p < 0.0001

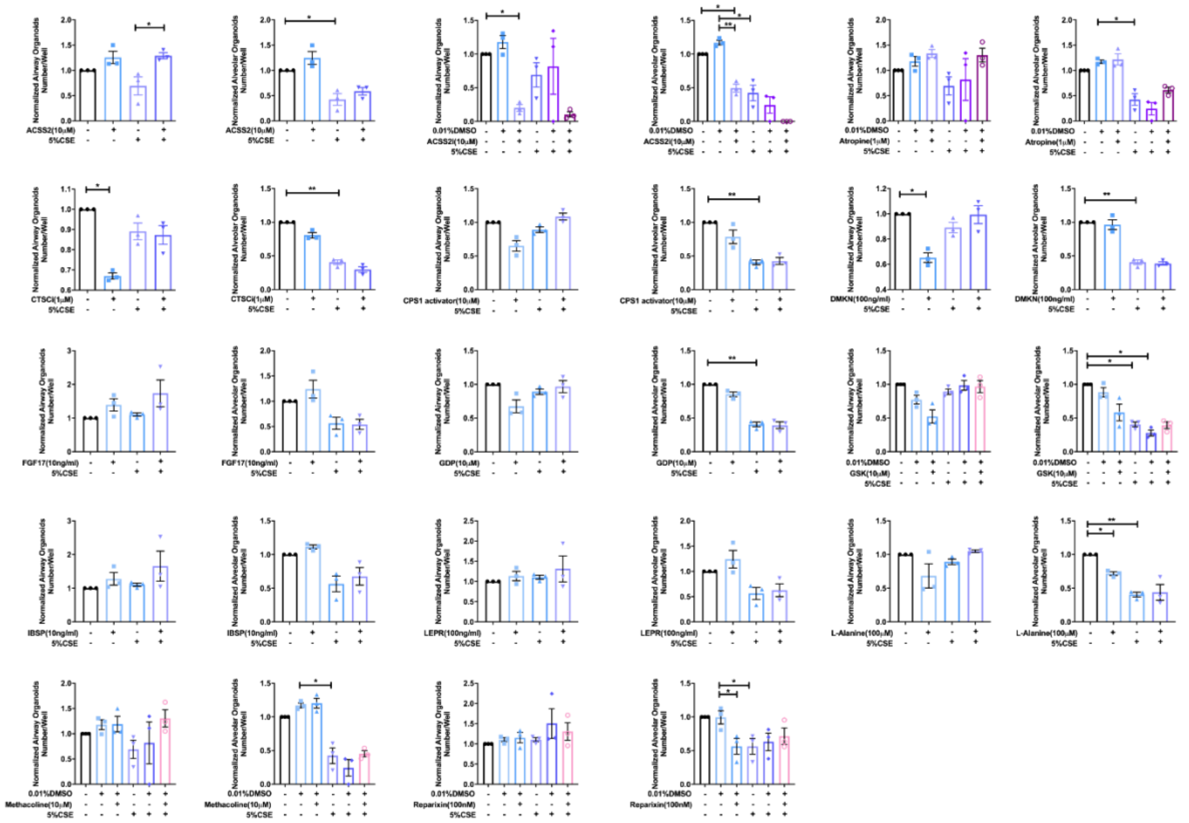

Fig. S2. Effects of 15 drugs candidates of interest (including an additional agonist and antagonist for one gene target) on the normalized number of airway and alveolar type lung organoids in the presence and absence of 5% CSE. Data are presented as median ± SEM. $**p < 0.05$, $**p < 0.01$, $***p < 0.001$, $****p < 0.0001$.

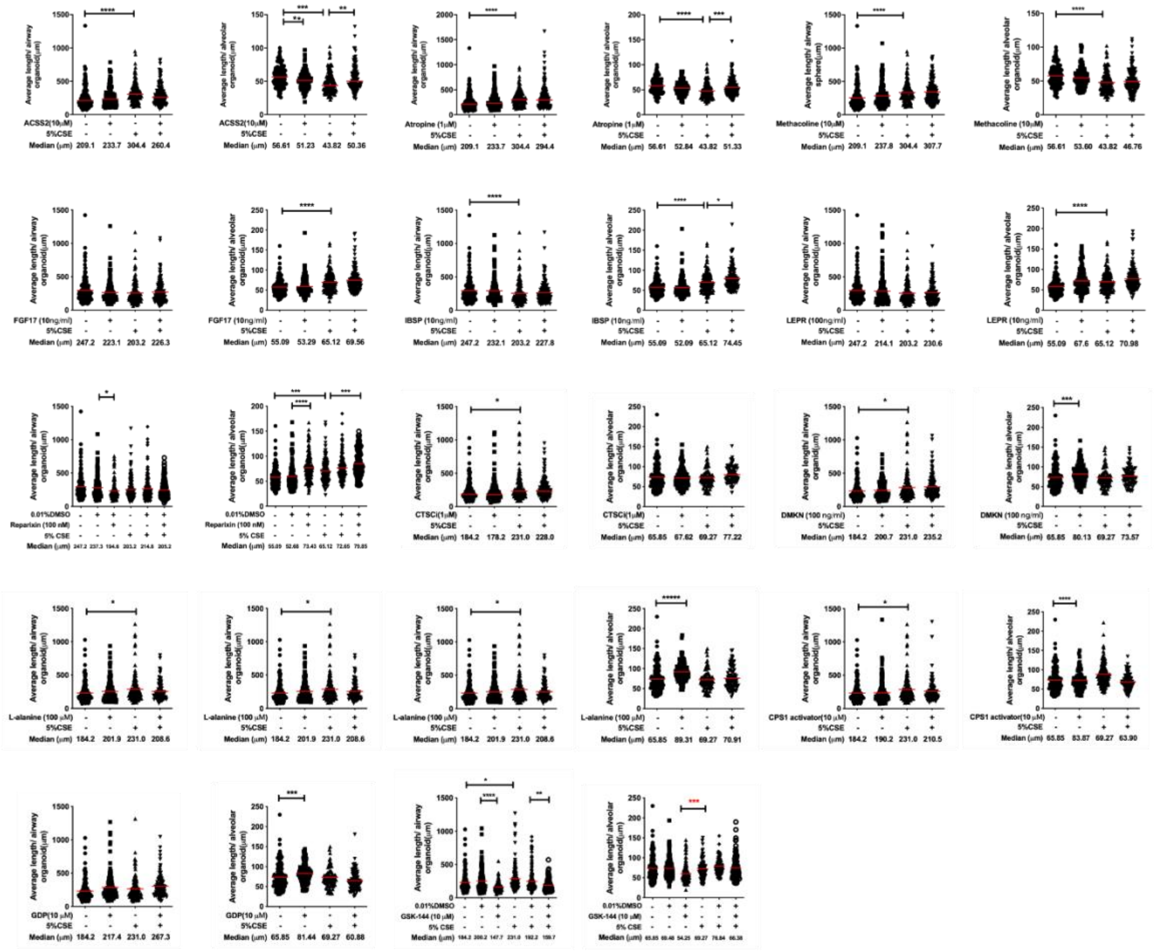

Fig. S3. Effects of 15 drugs candidates of interest (including an additional agonist and antagonist for one gene target) on the size of airway and alveolar type lung organoids in the presence and absence of 5% CSE. Data are presented as median ± SEM. **$p < 0.05$, **$p < 0.01$, ***$p < 0.001$, ****$p < 0.0001$.

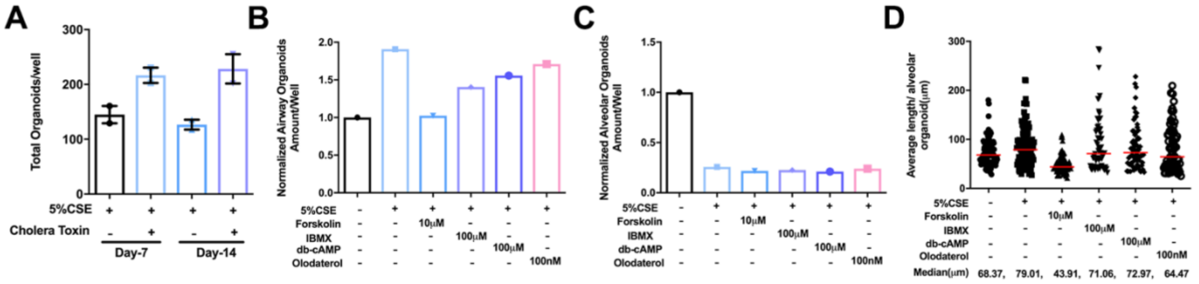

Fig. S4. Compounds related to cAMP signaling pathways tested on lung organoid assay. (**A**) Quantification of total organoids treated with 5% CSE + cholera toxin at different time points. (**B**) Quantification of normalized airway type organoids treated with 5% CSE ± Forskolin (10 µM), IBMX (100 µM), db-cAMP (100 µM), and olodaterol (100 nM). (**C**) Quantification of normalized alveolar type organoids treated with 5% CSE ± Forskolin (10 µM), IBMX (100 µM), db-cAMP (100 µM), and olodaterol (100 nM). (**D**) Quantification of average length (diameter) of alveolar type organoids (median value) treated with 5% CSE ± Forskolin (10 µM), IBMX (100 µM), db-cAMP (100 µM), and olodaterol (100 nM). Data are presented as median ± SEM. **p < 0.05, **p < 0.01, ***p < 0.001, ****p < 0.0001

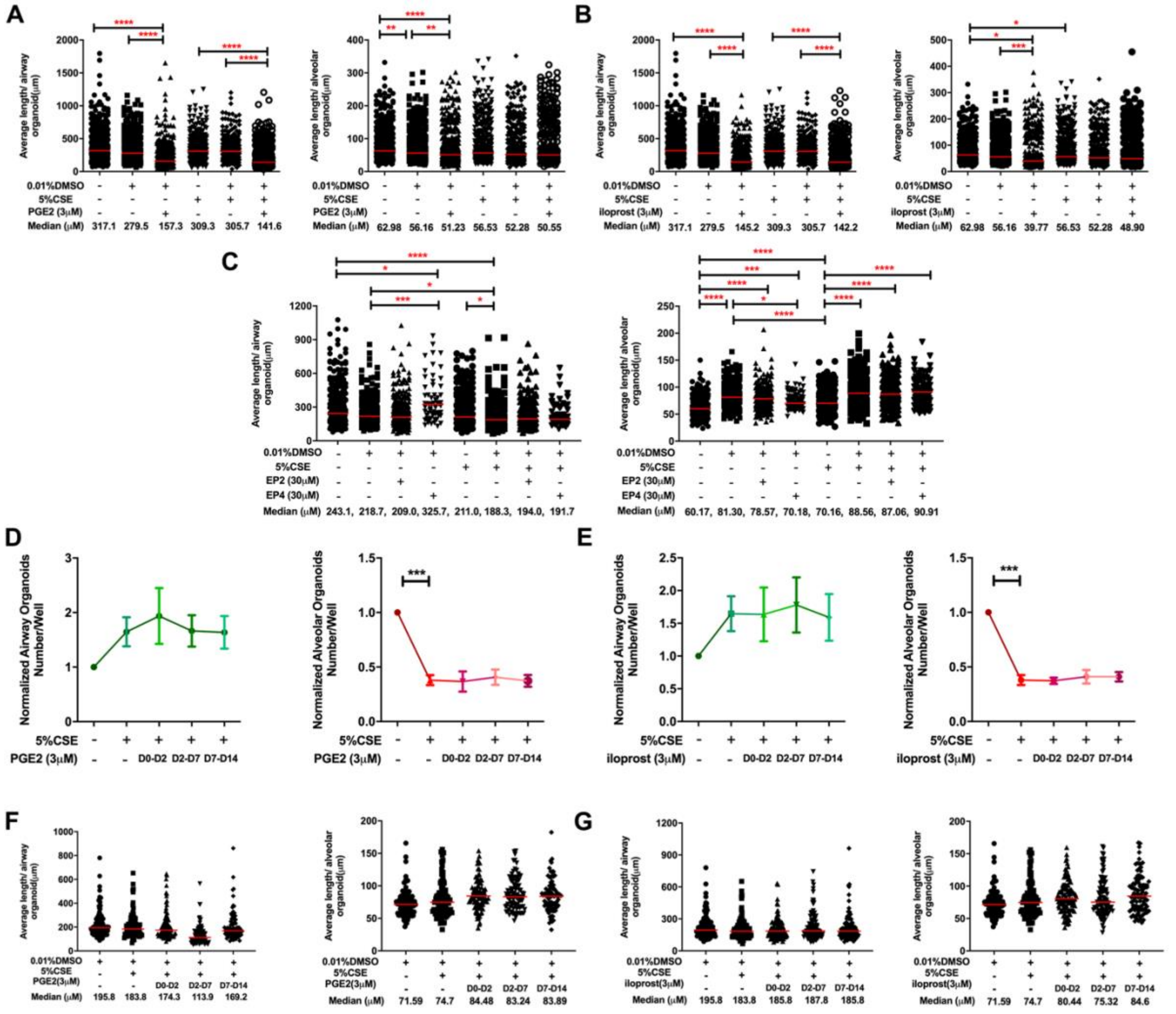

Fig. S5. Quantification of lung organoid numbers and sizes in the study of PGE2/PGI2/EP2/EP4. (**A-B**) Quantification of average length (diameter) of organoids (median value) of airway and alveolar type organoids treated with 5% CSE ± PGE2 agonist (A)/iloprost (B) measured on day 14. N = 5 experiments, n > 334 organoids/group. Data are presented as median ± SEM. (**C**) Quantification of average length (diameter) of airway and alveolar type organoids (median value) treated with 5% CSE ± EP2/EP4 agonists measured on day 14. N = 5 experiments, n > 264 organoids/group. Data are presented as median ± SEM. (**D-E**) Quantification of normalized number of airway and alveolar type organoids treated with vehicle control, 5% CSE ± PGE2 agonist (D)/ iloprost (E) from day 0-2, day 2-7, or day 7-14. (**F-G**) Quantification of average length (diameter) of airway and alveolar type organoids (median value) treated with 5% CSE ± PGE2 agonist (F)/ iloprost (G) from day 0-2, day 2-7, and day 7-14. N = 2 experiments, n > 85 organoids/group. Data are presented as median ± SEM.

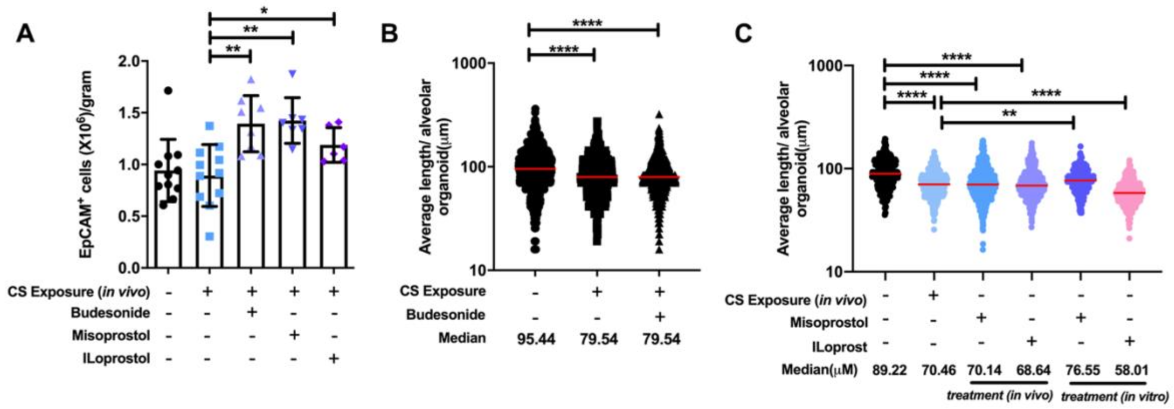

Fig. S6. Quantification analysis of lung organoid assay in the *in vivo* study of PGE2/PGI2/Budesonide. (**A**) Yield efficiency of Epcam+ cells from mice with different treatments. (**B**) Quantification of average length of alveolar type organoids co-cultured from CCL-206 and Epcam+ cells isolated from air- (control) or CS-exposed mice with or without *in vivo* treatment with misoprostol or iloprost (i.p injection). N = 4-8 experiments, n = 400 organoids/group. (**C**) Quantification of average length of alveolar type organoids co-cultured from CCL-206 and Epcam+ cells isolated from air- or CS-exposed mice. Organoids were treated *in vitro* with misoprostol or iloprost for 14 days. N = 4-8 experiments, n = 400 organoids/group. Data are presented as median ± SEM. **$p < 0.05$, **$p < 0.01$, ***$p < 0.001$, ****$p < 0.0001$.

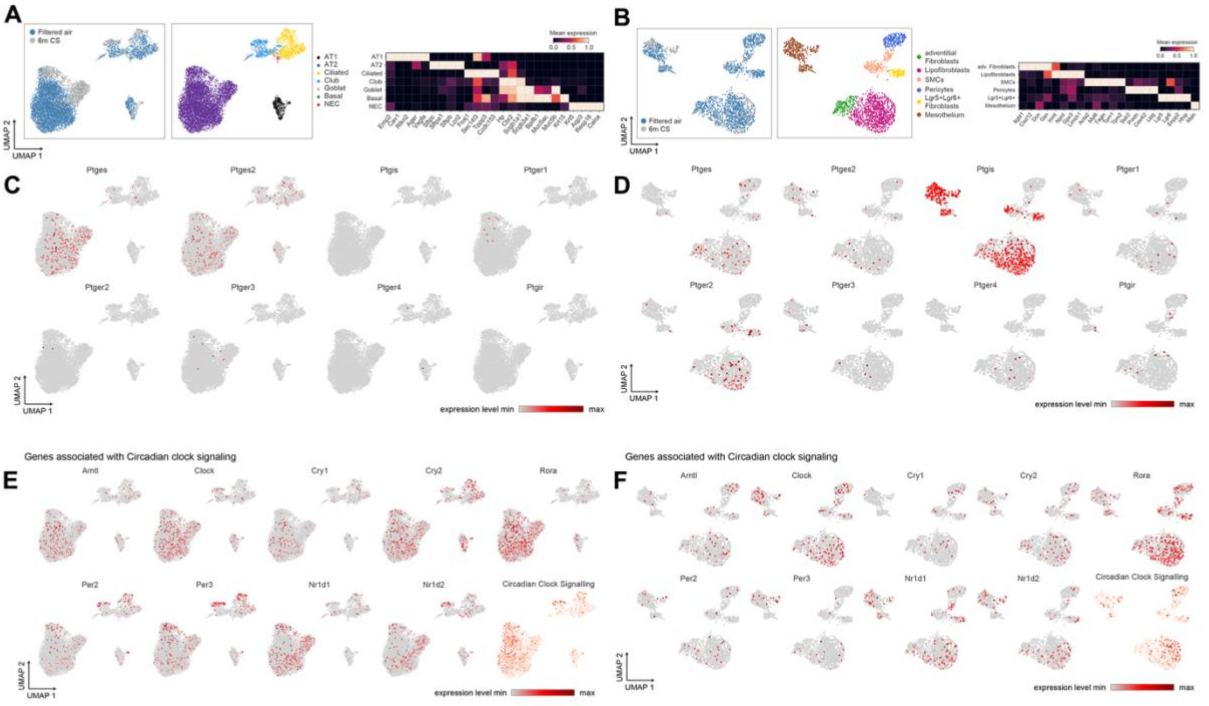

Fig. S7. sc-RNA-seq analysis of lungs from mice exposed to air and cigarette smoke (6 months) extracted from GEO151674. Data are extracted from the NCBI GEO database GSE151674. (**A**) UMAP of scRNA-seq profiles (dots) colored by air or cigarette smoke (6 months) in different epithelial cell types. (**B**) UMAP of scRNA-seq profiles (dots) colored by air or cigarette smoke (6 months) in different mesenchymal cells. (**C-D**) The cellular localization of expression *of Ptges, Ptges2, Ptgis, Ptger1, Ptger2, Ptger3, Ptger4* and *Ptgir* in epithelial cells and mesenchymal cells using scRNA-seq analysis of mouse lung tissue. (**E-F**) The cellular localization of *Arntl*

(Bmal1), Clock, Cry1, Cry2, Rora, Per2, Per3, Nr1d1, and *Nr1d2* in epithelial cells and mesenchymal cells using single cell RNA sequencing of mouse lung tissue.

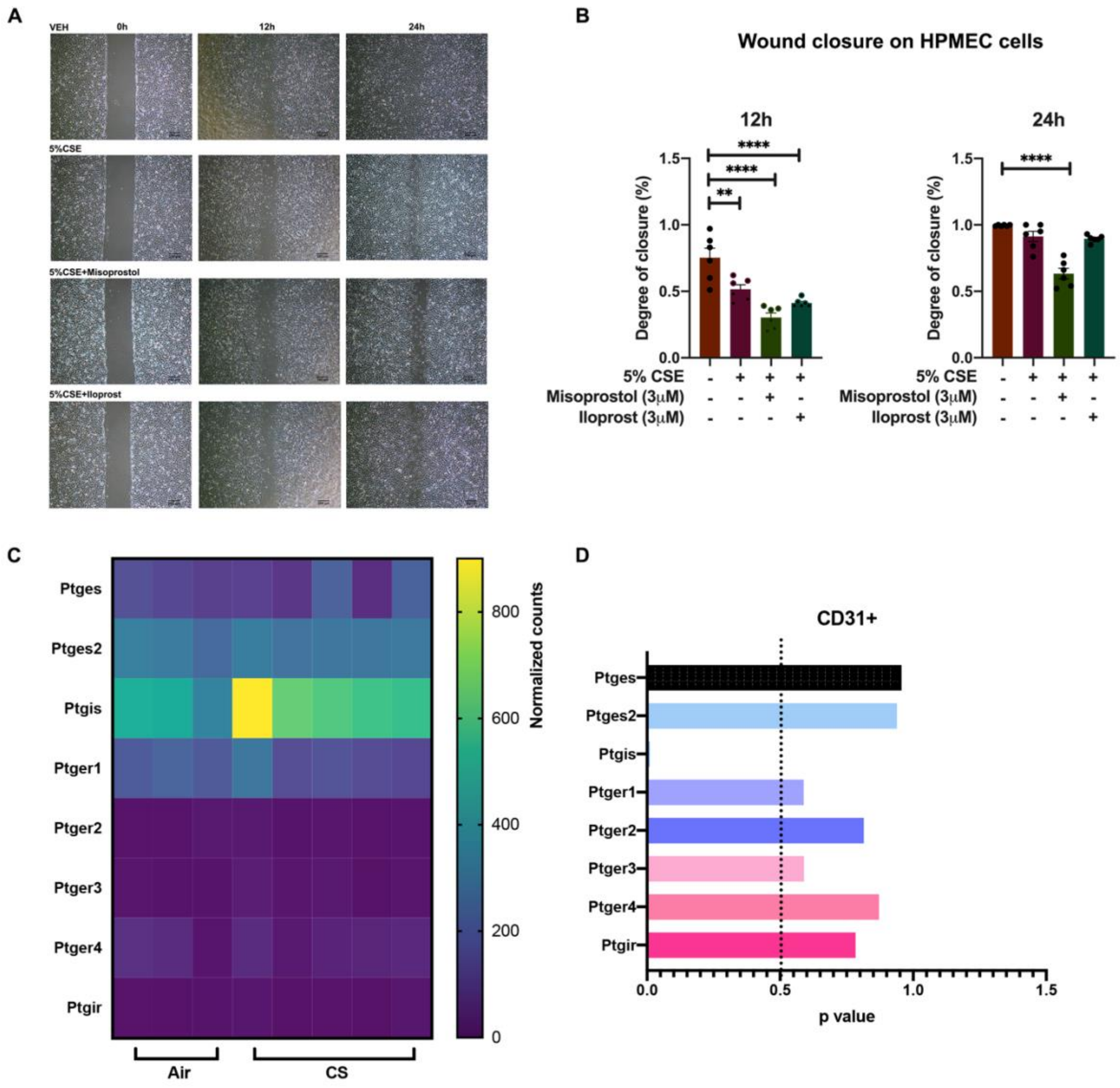

Fig. S8. PGE2 and PGI2 effects on pulmonary endothelial cells. (A). Representative images of HPMECs treated with 5%CSE ± misoprostol/iloprost (3μM) for 24h first and scratches were made afterwards, and images were captured every 12h. (**B)**. The degree of wound closure of HPMECs at 12h and 24h after scratch has been created. Data are presented as mean ± SEM. *p < 0.05, **p < 0.01, ***p < 0.001, ****p < 0.0001. (**C)**. Heatmap of normalized counts of Ptges2, Ptgis, Ptger1-4, and Ptgir in CD31+ cells from mice exposed to air or CS. (**D)**. P-values of the genes *Ptges, Ptges2, Ptgis, Ptger1-4*, and *Ptgir*.

Table S1. Information of final drug lists screened on organoid assay.

NO	Overlap Up	Full name	Drug/compound name	Final concentrations	Article No.	Company
**1**	CPS1	Carbamoyl-phosphate synthase 1	**N-Acetyl-L-glutamic acid**	10 µM	855642-25G	Sigma-Aldrich
**2**	CTSC	Cathepsin C/Dipeptidyl peptidase 1	Cathepsin c inhibitor	1 µM	BI-9740	Boehringer Ingelheim
**3**	CXCL1	C-X-C Motif Chemokine Ligand 1	Reparixin (L-Lysine salt)	100 nM	Cayman - 21492	Cayman chemical
**4**	DMKN	Derrmokine	Dermokine beta recombinant protein antigen	100 ng/mL	NBP1-86840PEP	Novus Biologicals
**5**	PTGIR	Prostaglandin I2 Receptor	Iloprost	0.03-, 0.3-, 3 µM	SML1651	Sigma-Aldrich
**6**	RAB8B	RAB8B, Member RAS Oncogene Family	CHEMBL384759/Guranosine 5'-diphosphate sodium salt (GDP)	10 µM	G7127	Sigma-Aldrich
**7**	TNNI3K	TNNI3 Interacting Kinase	GSK-114	10 µM		
**NO**	**Overlap Down**	**Full name**	**Drug/compound name**		**Article No.**	**Company**
**1**	ACSS2	Acyl-CoA Synthetase Short Chain Family Member 2	Acetyl coenzyme A sodium salt	10 mM	A2056-1MG	Sigma-Aldrich
**2**	ACSS2i		ACSS2 inhibitor	10 mM	S8588	Selleckchem.com
**3**	CHRM3	Cholinergic Receptor Muscarinic 3	Methacholine	10 µM	A2251	Sigma-Aldrich
**4**	CHRM3i		Atropine	1 µM	A0132	Sigma-Aldrich
**5**	FGF17	Fibroblast Growth Factor 17	Recombinant human FGF-17 protein	10 ng/mL	319-FG-025	R&D systems
**6**	IBSP	Integrin Binding Sialoprotein	Recombinant Human IBSP/Sialoprotein II Protein, CF	10 ng/mL	4014-SP-050	R&D systems
**7**	LEPR	Leptin Receptor	Recombinant Mouse Leptin Protein, CF	100 ng/mL	L4146-1MG	Sigma-Aldrich
**8**	PTGES2	Prostaglandin E Synthase 2	16,16-dimethyl Prostaglandin E2	0.03-, 0.3-, 3 µM	D0160	Sigma-Aldrich
			Misoprostol	3 µM	M6807	Sigma-Aldrich
**9**	SLC16A3	Solute Carrier Family 16 Member 3	Streptozocin	10 µM	S0130-50MG	Sigma-Aldrich
**10**	SLC1A4	Solute Carrier Family 1 Member 4	L-Alanine	100 µM	A7627-1G	Sigma-Aldrich

Table S2. Information of compounds used in organoid assay.

Name	Final concentrations	Article No.	Company
Rolipram	1-, 10 µM	R6520	Sigma-Aldrich
Budesonide	1-, 10-, 100 nM	B7777	Sigma-Aldrich
Cholera toxin	0.1 mg/mL	C8052	Sigma-Aldrich
(R) -Butaprost (EP2 analogue)	30 µM	B6309	Sigma-Aldrich
EP4 analogue*	30 µM	CAY10598	Cayman chemical
Forskolin	10 µM	F6886	Sigma-Aldrich
IBMX (3-Isobutyl-1-methylxanthine)	100 µM	Obtained from AppliChem (Germany)	
db-cAMP (Bucladesine)	100 µM	D0627	Sigma-Aldrich
Olodaterol	100 nM	Obtained from AppliChem (Germany)	

*EP4 analouge: 5-[(3S)-3-hydroxy-4-phenyl-1-buten-1-yl]1-[6-(2H-tetrazol-5R-yl)hexyl]-2-pyrrolidinone

Table S3. Top 20 pathways enriched in Epcam+ cells.

Sheet 1-6 show the top 20 pathways significantly up- and down regulated reactome pathways enrichment from differentially expressed genes within the comparisons of air vs CS exposure, CS exposure vs CS+misoprostol, and CS exposure vs CS+iloprost.

Data Set S1. Raw gene counts list for Epcam+ cells from mice exposed to air or CS.

Data Set S2. Raw gene counts list for Epcam+ cells from mice exposed to CS or CS+misoprostol (i.p.).

Data Set S3. Raw gene counts list for Epcam+ cells from mice exposed to CS or CS+ilprost (i.p.).
